# Performative planning creates a values mismatch between wildfire plans and community needs

**DOI:** 10.1073/pnas.2521536123

**Published:** 2026-04-06

**Authors:** Nicola Ulibarri, Ryan J. McCarty, Matthew Hamilton, Holly K. Nesbitt, Matthew A. Williamson

**Affiliations:** ^a^Department of Urban Planning and Public Policy, University of California, Irvine, CA 92697; ^b^Dry Scientific, Inc., Irvine, CA 92617; ^c^Haub School of Environment and Natural Resources, University of Wyoming, Laramie, WY 82072; ^d^Human-Environment Systems, Boise State University, Boise, ID 83706

**Keywords:** wildfire management, plan evaluation, Community Wildfire Protection Plans

## Abstract

To mitigate the potential impacts of wildfire, communities across the United States are engaging in collaborative wildfire risk mitigation planning. Planning involves identifying a set of goals, developing management strategies to achieve those goals, and codifying the goals and strategies in a written document. A plan’s goals and strategies are informed by values—the things plan authors and communities want to protect or enhance. Identifying and evaluating these values can give insight into whether a plan is meeting the needs of local wildfire risk and vulnerability, incorporating best practice from national policies, or simply reflecting broader cultural trends. This paper explores these tensions using the case of Community Wildfire Protection Plans (CWPPs), which are local (neighborhood to multicounty) plans developed collaboratively by diverse wildfire-related stakeholders. Drawing on a combination of manual coding and computational text analysis, we first characterize the values to be protected in 2268 CWPPs from across the United States. We then evaluate what shapes the values articulated in individual CWPPs, exploring the degree to which values reflect local wildfire risk and socioeconomic vulnerability, updates in policy guidance from US federal agencies, and/or broader cultural trends. This paper makes three primary contributions: 1) mapping local wildfire values at the national scale; 2) understanding tensions in environmental governance in a multilevel federalist system; and 3) advancing values as an important metric for evaluating the quality of management plans.

## Significance

As the size and intensity of wildfire in the United States increases, managing wildfire will require addressing multiple interconnected impacts. Most Community Wildfire Protection Plans (CWPPs) prioritize the built environment and human health, with less attention to wildfire’s impacts on the economy, natural environment, or cultural services. Plans do not reflect the unique circumstances of wildfire vulnerability in a specific place, nor do they incorporate new federal policy updates. At best, they reflect ideas discussed in broad popular discourse. Community planning for wildfire across the United States is not aligned with wildfire risk or federal guidance; communities remain vulnerable to wildfire because best practices for effective planning are not being followed.

Managing wildfire is challenging. US federal agencies spent an average of $2.9 billion annually on wildfire suppression between 2019 and 2023 (1), with state, local, and private entities also spending a significant (uncounted) amount. Nonetheless, losses from fires have increased steadily (2) alongside the size and intensity of fires (3–5). As wildfire risk management practitioners increasingly recognize the need for new and adaptive approaches for addressing wildfire (6, 7), communities across the United States are engaging in wildfire planning. Planning involves identifying a set of goals, developing management strategies to achieve those goals, and codifying the goals and strategies in a written document (8, 9). A plan’s goals and strategies should reflect a community’s priorities and values; indeed, the American Institute of Certified Planners code of ethics dictates that planners “Identify social and cultural values which should be preserved as well as natural elements” (10). In community wildfire plans, which are often not written by professional planners, these priorities are often expressed as “assets,” “values to be protected,” or “values at risk” (hereafter “values”), which are concrete things a plan aims to protect or enhance (11).

This paper analyzes the values stated in community wildfire plans. Understanding the things people impacted by wildfire want to protect is critical for designing management approaches that match those values (12, 13) and is an important component of plan quality (14). The scope of values articulated in a plan can also provide insight into how wildfire is being managed and potential gaps in selected protection strategies (15). The way a policy problem is framed often dictates what management approaches are considered (16, 17), and there are numerous potential narratives about the causes and consequences of wildfire (18, 19). If a plan focuses only on protecting infrastructure while not mentioning public health, there is likely less attention paid to preventing smoke exposure or other health impacts, suggesting a need for additional policies or management actions if health becomes a policy priority. Without recognizing the complex, multidimensional nature of wildfire, plans risk developing mitigation strategies that overlook key impacts or populations (20–22).

What comprises a “good” set of values? In a federalist system like the United States, there are multiple potential sources of plan content: Plans may reflect local needs and discretion, or they may reflect higher-level policies or incentives. Each source of influence comes with strengths and weaknesses. The concept of social–ecological fit argues that governance structures are most effective when they match the scale and context of a specific social–ecological system (23–25). To achieve social–ecological fit, values should match the specific types of wildfires that occur and characteristics of the impacted communities in a particular place. For instance, a community with an economy driven by logging should express different values to be protected than a community with a large population of retirees (26). If both places developed plans based on the same values, the proposed management strategies might not be tailored to their specific needs and therefore may be ineffective at protecting the community.

At the same time, including values that reflect larger-scale priorities like those set by national governments can have strategic advantages. In many countries, national government agencies have substantially more capacity than local communities (27–29). Their policy priorities are therefore better able to reflect the newest updates in science as well as address regional, national, or global scale problems that require larger collective action (e.g., climate change), and they can draw on more financial resources to support the implementation of such policies. Community-driven management may be slow to incorporate these changes, leading to slower transformation of the system overall.

Finally, rather than reflecting either local needs or national priorities, plans may simply reflect the general cultural imagination of the time they were written. Social movements, media discourse, and popular culture all shape what people are thinking and talking about (30, 31). As plans are written in a particular historical moment, the cultural zeitgeist of that time may shape the values and priorities encoded in the plans; for instance, a value may appear in a plan because the author read a newspaper article discussing that value. While the zeitgeist can reflect widely held concerns, these concerns may not reflect the needs of specific places nor the particularities of wildfire.

This paper explores these possible sources of plan values using the case of Community Wildfire Protection Plans (CWPPs) in the United States. CWPPs are local plans, developed for jurisdictions ranging from neighborhoods to multicounty areas through collaborative processes that bring together diverse wildfire-related stakeholders (32). CWPPs were introduced in the Healthy Forests Restoration Act of 2003. The primary objective of CWPPs is to enhance collaboration between local governments, state and federal agencies, and other stakeholders to reduce wildfire risk and improve forest health. CWPPs allow communities to identify and prioritize areas for hazardous fuel reduction treatments and recommend measures to reduce structural ignitability throughout the at-risk community. CWPPs also aim to strengthen emergency management preparedness and response, as well as promote community awareness and education about wildfire risks. CWPPs require approval by “the applicable local government, local fire department, and State agency responsible for forest management, in consultation with interested parties and the Federal land management agencies managing land in the vicinity of the at-risk community” (16 USC § 6511). In practice, the size and makeup of the group of participating organizations varies substantially across individual CWPPs, and the authors themselves can be fire departments, local governments, state forestry departments, or professional consultants (32). Once a community has a CWPP in place, they are eligible to apply for federal funding through the Community Wildfire Defense Grant (CWDG) Program. See SI Text for more details on the specific requirements and incentives associated with CWPPs.

The various sources of plan values also reflect concerns about performative planning, wherein plans are developed for tactical purposes rather than to solve a policy problem (8, 33, 34). Plans that only reflect higher-level requirements, rather than local context, may indicate that their authors are simply checking boxes as baseline compliance. Such plans may be less likely to be implemented and therefore affect wildfire resilience. As CWPPs are voluntary in many states, yet required for funding, tactical compliance may be common.

Drawing on a combination of manual coding and computational text analysis, we first characterize the values to be protected from a new dataset of 2268 CWPPs from across the United States. We then evaluate what shapes the values articulated in individual CWPPs, exploring whether values reflect local dynamics of wildfire risk and socioeconomic vulnerability, updates in policy guidance from US federal agencies, and/or broader cultural trends. This paper makes three primary contributions: 1) mapping local wildfire values at the national scale; 2) understanding tensions in environmental governance in a multilevel federalist system; and 3) advancing values as an important metric for plan quality evaluation.

## Results

### Most Plans Aim to Protect the Built Environment.

**Fig. 1.**
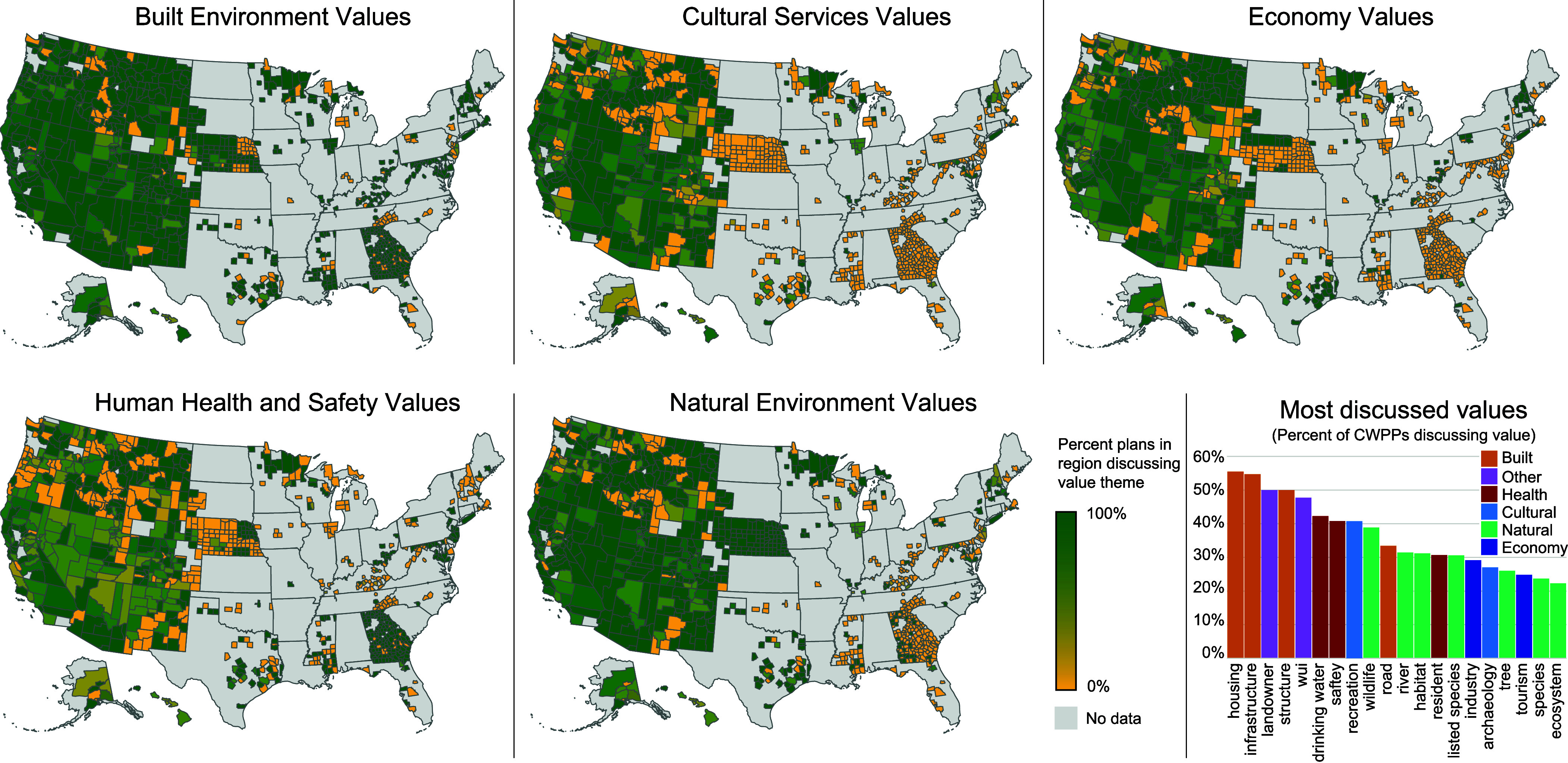
Maps showing the percent of plans in each county discussing each of the five value themes. Bottom right: Bar chart showing percent of CWPPs discussing each of the top 20 values. Orange = built environment; light blue = cultural services; dark blue = economy; maroon = human health and safety; green = natural environment; purple = other.

CWPPs highlight a diverse range of values to protect. Individual value topics were derived from the 1000 most common lemma (stemmed words and synonyms) in value-related text from the CWPPs (see *Materials and Methods*). This analysis identified 64 individual values, with several nested (e.g., oak as a subset of tree). *SI Appendix*, Table S1 provides the full list of values, associated keywords, and relative frequency, and Fig. 1 shows the top 20 most discussed terms. The most common values are *housing* (55.5% of CWPPs that discussed any values^*^), infrastructure (54.7%), landowners (50.0%), and structures (50.0%). Some CWPPs include fairly unique values to protect, including specific plant and animal species [e.g., eagle (4.9%), salmon (2.8%)], specialized infrastructure [e.g., hospital (6.7%), cabin (5.6%), irrigation (4.5%)], and unique economies [e.g., ski (4.4%)]. Interestingly, some relatively uncommon values are terms one might expect to be used more frequently given the context of wildfire, such as logging (15.4%) and firefighter (12.8%).

Individual values were aggregated into five themes. Built Environment was the most discussed theme, with 88.2% of plans identifying at least one value related to the built environment. Built Environment has strong geographic coverage across most counties where plans are present (Fig. 1). Built Environment is also consistently the top theme in each plan publication year (*SI Appendix*, Fig. S1). 74.3% of CWPPs discussed Human Health & Safety, with the most consistent coverage in Arizona, California, Georgia, New Jersey, and Maryland. 67.5% discussed Natural Environment, with consistent coverage across the southwestern United States as well as Minnesota and Nebraska. 53.8% discussed Economy, with most consistent coverage in California, Montana, Nevada, New Hampshire, and Utah. Finally, 51.3% discussed Cultural Services, with most consistent coverage across Nevada and Utah.

Looking at changes in topic frequency over time, most of the top 20 topics remain steady or decline slightly over time. Industry, tourism, and wildfire all peak just before and into 2020, but decline overall. Infrastructure is the only top-20 value increasing over time. Across all values, there is a significant decline in the number of individual values mentioned over the 22-y period of observation, with the average plan discussing 0.2 fewer values each year.

### Values Reflect a Plan’s State More Than Local Wildfire Context.

We next explore whether a plan’s local context influences its likelihood of discussing each value theme (see Table 1 for a summary of significant results and *SI Appendix*, Tables S2–S6 for full model results). The only consistent influence of any context factors is on Human Health & Safety, as CWPPs from counties with lower fire risk but more exposed structures, higher hazard potential, and more federal workers are more likely to prioritize health. The effects of all other variables are not consistently nonzero (pd < 90%) when state fixed effects are included, which means that discussion of values is determined more by what state a plan is located in than by local context. However, looking at the models without state fixed effects highlights the contexts under which each value theme is discussed.

**Table 1.** Local context variables that are significantly related to each value theme

Theme	Wildfire risk	Hazard exposure	Socioeconomic vulnerability	Social capital
Built				
Cultural	+ Fire_Intense − Exposure	+ ALR_VALA, ALR_VALB	+ asthma, phone − poverty, houseburden, language	+ charitable, educ_eq, income_eq − union, state, employ_eq, gender_inc
Economy	− Exposure	− PM25	+ asthma, expect, transport, phone − poverty, houseburden, language	+ educ_eq, ethnic_eq − union, over18
Health	**+ Exposure, WHP** **− Fire_Risk,** Fire_Intense	+ ALR_VALA − ALR_VALB	+ unemployed, plumbing, language − asthma, transport	+ union, **federal**, income_eq, civic_ops − ethnic_eq
Natural	+ Fire_Intense − Exposure, WHP	+ ALR_VALA − PM25	+ income, expect, transport, phone − houseburden	+ charitable, local, educ_eq − union, employ_eq, gender_inc, under65, politic_act, over18

Note: Bolded variables are consistently nonzero (pd > 90%) after state fixed effects are added.

Counties with more intense fires but lower exposure are more likely to discuss Cultural Services and Natural Environment, and those with lower exposure are more likely to discuss Economy. Interestingly, no wildfire variables influence prioritization of the Built Environment. Values also reflect a county’s experience with natural hazards in general, with a county’s annual agricultural loss rate positively influencing Built Environment, Human Health & Safety, and Natural Environment. Counties with lower PM 2.5 pollution are more likely to discuss Economy and Natural Environment.

Turning to social vulnerability, Cultural Services are more frequently discussed in counties with less poverty, more chronic asthma, lower housing burden, higher access to landline phones, and a lower fraction of English speakers. Economy is discussed more frequently in counties with more asthma, a higher life expectancy, better access to transportation and telephones, lower poverty, lower housing burden, and fewer English speakers. Human Health & Safety is more likely to be discussed in counties with higher unemployment, more households lacking indoor plumbing, lower asthma rates, a higher fraction of English speakers, and lower transportation access. Finally, Natural Environment is more likely to be discussed in counties with higher household income, higher life expectancy, lower housing burden, higher transportation access, and higher access to landlines.

Regarding social capital, Cultural Services are discussed in counties with more charitable organizations, more educational and income equality, less union participation, less state employees, and lower gender and employment equality. Economy is discussed more in counties with higher educational and ethnic similarity, fewer unions, and more children. Human Health & Safety is discussed more in counties with more unions and federal workers, higher income equality, higher civic opportunities, and lower ethnic similarity. Natural Environment is discussed more in counties with more charitable organizations, more local government employees, higher educational similarity, fewer unions, lower employment and gender equality, less politically active residents, and more residents over 65.

### Some Federal Policies Shape Plans, but Plans also Shape Policies.

**Fig. 2.**
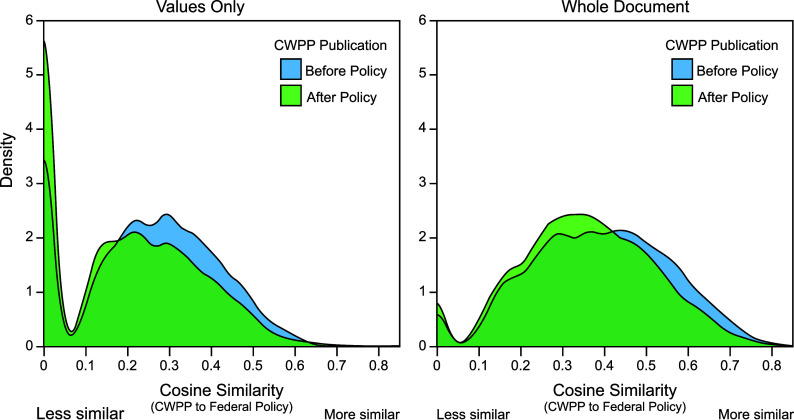
Density plots of cosine similarity between CWPPs and federal policies, comparing plans published before policies were adopted and after. *Left*: comparing only values terms stated in policy goals or objectives section; *Right*: comparing values terms stated in the entire policy text.

We next evaluate similarity between values stated in CWPPs and those stated in federal wildfire-related policies by calculating cosine similarity between each plan and policy. Across all plan-policy combinations, mean cosine similarity is 0.296, with a minimum of 0 and maximum of 0.846 (where 1 indicates perfect alignment of included and excluded values in the two compared documents). Average similarity to federal policy is higher for plans published *before* a policy was adopted, compared to those published after (0.318 vs. 0.278). This trend holds whether comparing CWPPs to the specific values and objectives of each policy, or to the entire policy document (Fig. 2).

According to the regression results (*SI Appendix*, Table S7), CWPP values are most likely to have any similarity (the zero-inflation portion of the model) to the values stated in the Community Forest Restoration Act, with all other policies having a negative or nonsignificant relationship. Given some overlap in values (similarity > 0), CWPP values most closely match the National Cohesive Wildland Fire Management Strategy (Cohesive Strategy), Forest Landscape Restoration Act (FLRA), Wildfire Crisis Implementation Plan (WCIP), and Stewardship End Result Contracting.

Comparing changes in similarity before and after a policy was enacted (the Timing*Policy interactions in *SI Appendix*, Table S7), similarity increases significantly after policy adoption for only two policies: The Cohesive Strategy and WCIP. CWPP similarity with the FLRA and Stewardship End Result Contracting both decrease significantly after adoption, with the remaining policies being nonsignificant.

Last, CWPPs written at the Community level have lower average similarity scores relative to County plans. Plan jurisdiction does not alter a plan’s receptiveness to policy change, as Timing*Level interaction terms are all nonsignificant.

### Economic Values Derive Most Strongly from Cultural Zeitgeist.

**Fig. 3.**
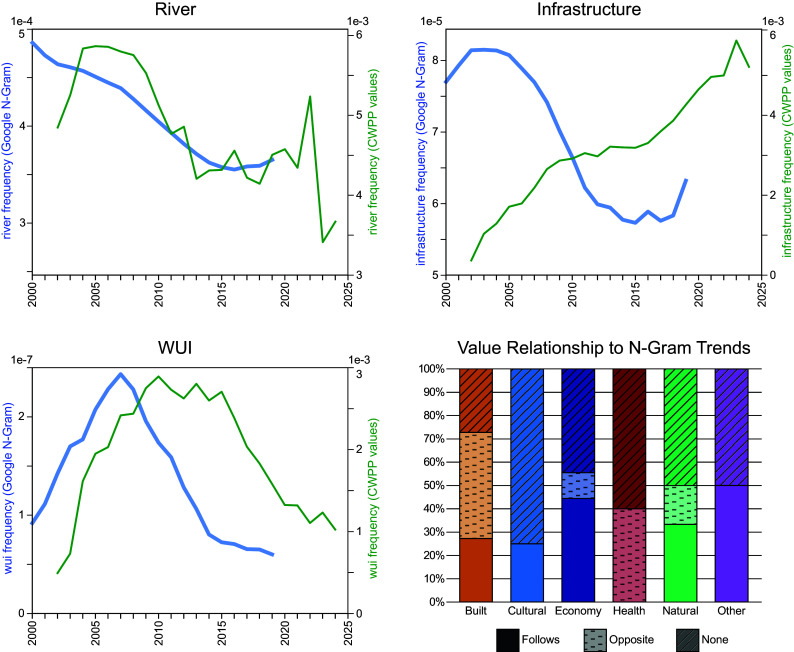
*Top Left*: Discussion of “River” in CWPPs “follows” the frequency of usage in the Google N-gram dataset. *Bottom Left*: “WUI” discussion in CWPPs follows N-gram usage, with a 2 to 3 y lag. *Top Right*: “Infrastructure” discussion in CWPPs is “opposite” the N-gram frequency. *Bottom Right*: A bar chart showing the percentage of individual values within each theme that follow, oppose, or are not related to the N-gram data.

Finally, we compare CWPP values with each term’s temporal frequency in the Google N-gram dataset (35) as an indicator of the terms’ general popularity in written media. 31.4% of the values articulated in CWPPs are positively and significantly correlated with the general frequency of those terms in the Google N-grams, meaning their use could be attributed to the overall cultural popularity of the term/idea, rather than to a specific need related to wildfire. This includes terms that have a strong positive correlation in the year the CWPP was published, as well as terms that saw a 1- to 3-y delay between their popularity in the general public and their usage in CWPPs (e.g., Fig. 3, *Top* and *Bottom Left*). 25.5% of values were negatively correlated with the zeitgeist (e.g., Fig. 3, *Top Right*), and 43.1% had no significant relationship.

Across the five value themes, Economy was most likely to reflect the general zeitgeist, with 44% of its underlying topics being positively and significantly correlated to their associated N-gram frequency, followed by Natural Environment (33%), Built Environment (27%), and Cultural Services (25%). In contrast, none of the topics comprising Human Health & Safety were positively correlated with the Google N-grams, and 40% had the opposite valence (Fig. 3, *Bottom Right*).

In addition, 16 of the value topics (25%) saw their correlation increase when adding a lag between the N-gram data and the CWPP publication date. The average correlation was highest after 2 y, meaning that CWPPs most closely reflect the cultural zeitgeist from 2 y prior to their publication.

## Discussion

Our analysis finds that CWPPs collectively articulate diverse values to protect, with primary emphasis on the built environment and human health and safety. These are arguably the two most “direct” impacts of wildfire for human populations and are therefore understandable priorities. However, only half to two-thirds of plans discussed values related to wildfire’s interconnected impacts on the economy, natural environment, or cultural services, with each proportion being an underestimate since we excluded plans that did not mention any values. Ignoring these impacts could degrade a community’s long-term resilience. A healthy, biodiverse landscape can result in less intense fires (36) with less smoke (37), helping to curb the likelihood that the fire threatens buildings or human safety. And wildfires can have long-term impacts on a community’s economic growth and recovery (38–40). While these are local problems, the fact that plans are undervaluing the economy, natural environment, and cultural services at scale could also result in aggregate impacts for the country.

The decline over time in references to most values (aside from infrastructure) suggests that CWPP authors are focusing on an increasingly narrow set of values. This could reflect terminology shifts or grouping of values into larger classes (e.g., that earlier plans would talk about both “habitat” and “species” while later plans only discuss one). At the same time, the fact that many terms decline in frequency in the Google N-gram dataset suggests that these shifts may be due to ideas going out of fashion in the cultural zeitgeist.

Contrary to best practices for good governance, rather than reflecting either local context or national policies, CWPP values are highly correlated within states and in some instances mirror the cultural zeitgeist. The effects of almost all local context variables become negligible when adding state-level fixed effects, suggesting that a driving force is state-level guidance about what to include in a CWPP, rather than the local (county-level) context. Indeed, some states (e.g., Georgia, Utah) encourage the use of state-specific CWPP templates that dictate discussion of particular values. In Georgia, the template emphasizes public safety and protecting homes and infrastructure. As a result, as Fig. 1 shows, most Georgia plans talk about Human Health and Built Environment, with less consistency discussing the other value themes. State-level clustering of values could also result from the influence of individuals who assist in the preparation of numerous plans. Prior research has demonstrated that patterns of cross-plan author participation are themselves clustered within states (41).

Characteristics of wildfire risk, intensity, and exposure—which we assume would shape people’s wildfire concerns—only consistently influenced discussion of Human Health & Safety. While socioeconomic vulnerability does play a role in shaping which values were discussed, we found (essentially) that only wealthier communities with better infrastructure prioritize values beside health. The social capital context suggests that bridging, bonding, and linking social capital all shape the values prioritized, but these capitals can both facilitate and block inclusion of particular values. For instance, Natural Environment is discussed more in places with more charitable organizations and local government employees but fewer politically active residents, which suggests that vocal residents may restrict environmental protection.

Regarding policy similarity, we find that while some federal policies lead to changes at the local level, just as often federal policies seem to adopt values already prominent in local-level planning processes. The two policies that did show an increase in CWPP similarity after adoption—the Cohesive Strategy and the WCIP—are both guidance documents that were developed by federal agencies with extensive community involvement over multiple years. For instance, the Cohesive Strategy says, “Regional and local stakeholders have been involved—they have had a seat at the table—and their valued perspectives brought the national wildland fire management decision-making process to a new level” (6). This engaged approach may have led to broader awareness of and buy-in to these policies, and therefore increased uptake of their ideas in subsequent CWPPs. In contrast, we observed no instances of federal legislation (passed by Congress) translating into new values at the local level. This mirrors broader trends that have been observed in political science, where changes in public opinion tend to precede adoption of policies by the federal government (42, 43).

Finally, we observe a significant correlation between the broader cultural zeitgeist and almost one-third of the values discussed in CWPPs, especially those related to the economy and natural environment. This suggests that CWPP authors talk more about (for example) the “wildland–urban interface” because it was a popular topic in written media at the time they were preparing the CWPP—or more specially, 2 y prior to when the CWPP was published.

While our analysis cannot determine which source (local context, federal policy, or cultural zeitgeist) has more or less influence on CWPP values, we can conclude that there is not a strong signal from either local context or federal policy (except policies developed collaboratively with communities). While institutional fit is only one determinant of successful environmental management (44), the lack of fit observed in the case of CWPPs suggests that communities are developing “one-size-fits-all” plans that may be ignoring uniquely vulnerable populations, important economic sectors, or valuable ecological sites—at the same time that plans are not incorporating new science or best practices promulgated by the federal government. Such baseline compliance makes CWPPs appear to be prepared in order to access funding, signaling their use as more performative rather than substantive plans. However, given that the United States is arguably not preventing nor recovering well from wildfire, improving the “fit” of CWPPs such that they become more substantive plans may be worth trying.

Limitations to our analysis provide fruitful avenues for future research. First, we do not have access to state-level CWPP guidance or templates; to fully understand why states have such a strong influence would require assessing this guidance. Second, we measure values using the language embedded in plans, but these may not directly reflect the way members of a community would speak about their preferences. Comparing plan values to the values people themselves hold (measured through a survey of residents or decision-makers) could provide better insight into synergies and gaps. Finally, we only measure the most frequent values (those present in the most common 1000 lemma); some communities may hold unique values that are not captured in an aggregate assessment.

Several policy recommendations stem from this research. First, prioritization of the built environment and human health may stem from the CWPP process being largely technocratic, where individuals or organizations with forest or fire expertise do the bulk of design and implementation (45). While these individuals have extensive expertise in how to protect things, deciding what to protect may require broader participation by residents, businesses, nonprofits, and other stakeholders. Second, the clustering of values at the state level suggests the state as a fruitful point of intervention to improve plans. Helping states develop more flexible guidance, with alternative sections tailored for specific communities or wildfire risks, could result in better recognition of local needs. Assuming diffusion of ideas through author networks, similar outcomes could be achieved by providing training for consultants and other frequent plan authors on tailoring approaches and recognizing policy updates. Federal agencies promulgating best practices and nonprofit organizations seeking to improve wildfire resilience may find added influence by coordinating closely with the states to support the development of future CWPPs. Third, the fact that many values appear more frequently in less socially vulnerable counties suggests that state or federal agencies—alongside their nongovernmental partners—should help ensure that lower-income, health-burdened communities are able to protect their economies, cultural services, and natural environments from wildfire. The CWDG (CWDG) program, launched in 2022 with approximately $1 billion in funding from the Infrastructure Investments and Jobs Act, directs significant resources to low-income communities in high-hazard areas. While the short period since this program’s inception limits efforts to measure its impact, the program presents a compelling model for supporting local efforts to protect a broad range of values at risk, and future programs should build upon the CWDG’s attention to the intersection between risk and vulnerability. Nongovernment organizations working in the wildfire planning arena may also strategically prioritize regions of the country with higher socioeconomic vulnerability for additional planning capacity (grants, staff support, training) using publicly available tools to determine program eligibility. Finally, local communities may benefit by expanding the scope of participation in wildfire planning processes beyond those immediately involved in fire and forest management to improve the likelihood that plans protect the broad suite of values that define the community as a place.

## Materials and Methods

### CWPP Collection and Processing.

We assembled a dataset of CWPPs from across the United States. Plans were collected through a combination of web searches and direct email and phone outreach to state agencies responsible for forest or wildfire management. We also incorporated 1062 plans collected for 11 Western states, hosted by FAC Net (46). While we endeavored for as comprehensive a dataset as possible, in some cases it is impossible to know whether we collected all plans that have been developed within a state. Some states maintain public repositories of available plans, some collect plans for internal use but do not make them publicly available, and some have no central record of locally developed plans. We ultimately collected the full document for 2268 CWPPs from 43 states. Of the remaining states, four have no plans and three have plans we were unable to access. For each plan, we recorded metadata including the plan location (state and county/counties), jurisdiction type (county, community, or fire protection district), and date published. *SI Appendix*, Fig. S2 shows the location of the plans by county and the number published by year.

There is no standard format for CWPPs, though some states have templates or guidelines that plans follow. Given this diversity, we used a word search approach to identify the plan section(s) where values were discussed. We searched the full plan text for values-related keywords (e.g., “values at risk,” “values at stake,” “values to be protected,” “values protected,” “values,” “assets at risk,” “goals,” “desired outcomes,” “features potentially at risk”). The final list of keywords was iteratively determined by reviewing where and how a sample of plans (27 plans from 11 states) discussed values. Some plans contained a dedicated section discussing values or objectives (titled, for instance, “Community Values at Risk,” “Assets at Risk,” “Values to be Protected”), while others had a few sentences or scattered the discussion across multiple parts of the plan. All values-related text for each plan was reviewed for relevance and copied into a database. 1882 plans (83%) contained text related to values (*SI Appendix*, Fig. S2).

To identify individual values, we used Natural Language Processing with heavy use of “humans in the loop.” We first used a text search query to find the 1000 most common lemma in the values-related text, where a lemma includes all stemmed versions of a word (e.g., river, rivers) as well as synonyms (e.g., stream, brook, creek). We reviewed this list to select items that could be considered “values” (omitting, for instance, verbs and adjectives, as well nouns that were vague like “land” or “area” or that were things to avoid like “erosion”). While the original list of values were derived from unigrams, in several instances, we added bigram (two-word) or trigram (three-word) search terms within a value to ensure specificity (e.g., “endangered species” vs. “endangered,” “Native American” vs. “native”). The full list of values and associated keywords is in *SI Appendix*, Table S1. Finally, we manually grouped the topics into five overarching themes: Built Environment, Cultural Services, Economy, Human Health & Safety, and Natural Environment. These themes were developed inductively from the individual values, and align with existing planning and assessment frameworks (e.g., ref. 47).

### Local Context.

To represent a plan’s local context, we use secondary data to measure wildfire risk, hazard exposure, socioeconomic vulnerability, and social capital. Wildfire risk includes the overall likelihood of a wildfire occurring, as well indicators for the intensity of a potential fire and its likely impact to structures (48). Hazard exposure includes the annual loss rate from any natural hazard for agriculture, buildings, and population (49). Socioeconomic vulnerability reflects the potential for a wildfire to have more extreme impacts on specific segments of the population, such as individuals with existing chronic health conditions or those who are unemployed. Relevant variables include economic indicators (e.g., income, poverty rates, and unemployment); health indicators (e.g., asthma, diabetes, life expectancy); and infrastructure access (e.g., transportation, telephone) (50). Finally, *social capital* reflects a community’s ability to access critical resources and knowledge through social relations. We include indicators for linking social capital (connections to government decision-makers), bridging social capital (connections with nonprofit and community-based organizations), and bonding social capital (relationships built on shared identity) (51). The full list of variables, descriptions, and data sources are in *SI Appendix*, Table S8.

We conducted a series of Bayesian logistic regressions (*SI Appendix*, Tables S2–S6) to understand whether a plan’s local context influences the likelihood of that plan discussing each of the five value themes. The outcome variable is a binary variable (presence or absence of the theme); predictor variables are the local context variables described above, as well as the plan jurisdiction (county, community, or fire protection district) and year published. We included all potential predictors in the model and used a horseshoe prior for regression coefficients. The horseshoe prior allows us to include all of the variables we considered plausible in determining the presence of a given value while also inducing sparsity in the final model to avoid overfitting (52). The general form of the model is

$$y∼Bern\left(p\right),$$

$$p=\frac{e^{\beta_{0}+\beta_{\mathrm{st}}+X\beta }}{1-e^{\beta_{0}+\beta_{\mathrm{st}}+X\beta }},$$

$$\beta_{0}∼N\left(0,2.5\right),$$

$$\beta_{\mathrm{st}}∼N\left(0,1\right),$$

$$\beta ∼N\left(0,\tau^{2}\lambda^{2}\right)\;\;(\mathrm{horseshoe}\;\mathrm{prior}),$$

$$\tau ∼\mathrm{Half}-t_{1}\left(0,\frac{0.2}{\sqrt{1}837}\right)\;\;(\mathrm{global}\;\mathrm{shrinkage}\;\mathrm{parameter}),$$

$$\lambda ∼\mathrm{Half}-t_{1}\left(0,1\right)\;\;(\mathrm{local}\;\mathrm{shrinkage}\;\mathrm{parameter}).$$

We fit a hierarchical Bayesian logistic regression for the occurrence of a value (y) with probability p based on a global intercept ($\beta_{0}$), a varying intercept for state ($\beta_{\mathrm{st}}$), and a vector of regression coefficients (β) multiplied by our various predictors (X). We used weakly informative priors for the intercepts given that support for the model is (0,1). We used a horseshoe prior to induce sparsity in the regression coefficients, similar to other regularized regression analyses (e.g., lasso or ridge regression). We set the global shrinkage parameter to reflect a prior belief that 20% of the predictors might provide information for the model given the total sample size (1837). The local shrinkage prior was a student-*t* distribution with one degree of freedom to enforce the horseshoe shape of the prior.

All predictor variables are estimated for the county where a plan is located; for plans that span multiple counties [*n* = 74 (3%)], we use the arithmetic mean of the involved counties.^†^ We use two model specifications for each outcome variable, one with just the predictor variables and one with fixed effects for the plan’s state.

### Policy Influence.

To understand the national policy context shaping plans, we reviewed literature on CWPPs, wildfire management, and forest management to identify major legislation, guidance documents, and federal agency strategies adopted during the period of observation. The list of selected federal policies is in *SI Appendix*, Table S9. For each policy, we used the same processing approaches as for CWPPs to identify values stated in the policy: 1) keyword searches to identify text related to each policy’s values and 2) whether any value topics were included in either the full text of the policy or in the values-related section of the policy.

To measure overlap of values between each federal policy and each CWPP, we calculated cosine similarity, a measure used to determine how similar two documents are to each other, based on the words they contain (53, 54). Cosine similarity treats each document as a vector in a multidimensional space, where each dimension corresponds to a unique word (or in our case, value topic) in the corpus. The similarity between two documents is calculated as the cosine of the angle between their corresponding vectors. The resulting similarity score ranges from 0 to 1, where 1 indicates perfect similarity (the two documents contain the same set of values) and 0 indicates no overlap of values. A unique similarity score was calculated for each plan/policy combination, resulting in 22,584 total scores.

To analyze the factors predicting the cosine similarity for each CWPP/policy pair, we employed a zero-inflated beta regression model. This statistical approach is particularly suited for our outcome variable (similarity), which ranges from 0 to 1 and includes a substantial number of zero values. The zero-inflated beta regression model combines two components: a beta regression for continuous values between 0 and 1 and a logistic regression for modeling the probability of observing a zero. The beta component models the continuous distribution of values between 0 and 1, capturing the inherent boundedness of proportion or rate data. Meanwhile, the zero-inflation component addresses the excess zeros that cannot be adequately explained by the beta distribution alone. This dual structure allows us to simultaneously account for factors influencing whether a response is zero and those affecting the magnitude of nonzero responses. Predictor variables for both the beta and zero-inflated component are Policy (a dummy variable for which federal policy a plan is compared to), Timing (whether the plan was published before “pre” or after “post” the federal policy was issued), and Level (a dummy variable for CWPP jurisdiction: county, community, or fire protection district). We include interactions between Policy*Timing and Level*Timing, to see whether plans published after a federal policy is released are more similar to that policy than those published before the policy was adopted, as well as whether certain types of plans are more responsive to federal policy changes. We present four model specifications: two with State (the state a plan was published in) as fixed effects and two as random effects; and two with the Policy*Timing interactions included in the zero-inflated component, and two with the interaction just in the beta component (*SI Appendix*, Table S7).

### General Culture.

To represent the general cultural zeitgeist, we use the Google N-gram dataset, also known as the Google Books N-gram Viewer (55). This dataset analyzes the frequency of words or phrases (n-grams) in books over time, using the large collection of books that Google has digitized as part of its Google Books project. As of 2019, it contained data from approximately 8 million books, or about 6% of all books ever published. While the database has some biases, such as overrepresentation of academic texts and variations in optical character recognition quality, by following documented best practices the N-gram dataset is a relatively reliable representation of general “popularity” of certain terms over time (56–58).

We compared word frequencies determined from our CWPP values dataset to word frequencies from the Google Version 20200217 N-gram dataset (the most recent version available for download, which includes the year 2019). The initial year (2001) and final year (2024) of the CWPP dataset were excluded from analysis due to low document counts. Using Pearson’s correlation coefficient, we compared correlation between plans and zeitgeist in the same year of CWPP publication, as well as 1-, 2-, 3-, and 4- y prior, in order to observe potential lags in influence. Based on the magnitude and direction of the correlation, each topic was categorized as “Following” the zeitgeist (positive correlation, *P* < 0.1), “Opposite” the zeitgeist (negative correlation, *P* < 0.1), or “None” (any correlation, *P* ≥ 0.1).

## Supplementary Material

Appendix 01 (PDF)

## Data Availability

Some study data available [Because some CWPPs cannot be shared publicly, the values text can be requested by contacting the corresponding author.). Previously published data were used for this work (48–51, 59).

## Footnotes

^*^The denominator for this and all following percentages is CWPPs that mentioned at least one value (*n* = 1882).

^†^To test sensitivity to the averaging approach, we also calculated a mean weighted by county land area. This did not affect the relative influence of any variable across models.
