# Supplementary material for: Performative planning creates a values mismatch between wildfire plans and community needs
Source: Proc Natl Acad Sci U S A. 2026 Apr 6;123(15):e2521536123. doi: 10.1073/pnas.2521536123 (PMC13079974; doi:10.1073/pnas.2521536123)
Supplement: Supplementary file 1 — Appendix 01 (PDF) [file pnas.2521536123.sapp.pdf]

PNAS

Supporting Information for

Performative planning creates a values mismatch between wildfire plans and community needs

Nicola Ulibarri[1,*], Ryan J. McCarty[2], Matthew Hamilton[3], Holly K. Nesbitt[4], Matthew A. Williamson[4]

1. Department of Urban Planning and Public Policy, University of California Irvine

2. Dry Scientific, Inc.

3. Haub School of Environment and Natural Resources, University of Wyoming

4. Human-Environment Systems, Boise State University

*Corresponding author: Nicola Ulibarri

Email: ulibarri@uci.edu

This PDF file includes:

Supporting text
Figures S1 to S2
Tables S1 to S9

Supporting Information Text

Overview of Community Wildfire Protection Plan Requirements and Incentives

Community Wildfire Protection Plans (CWPPs) are collaborative planning documents enabled by the Healthy Forests Restoration Act (HFRA) of 2003. Prior to HFRA, wildfire representatives at federal and state agencies, as well as local fire departments, bore primary responsibility for wildfire mitigation. HFRA provided a mechanism to encourage a broader suite of actors to participate in community-level planning for wildfire mitigation. As a planning model, CWPPs are quite flexible (1). The few requirements include that local and state governments must collaborate to develop these plans (along with federal agencies and other parties as appropriate), the plans must describe areas and methods for prioritized fuel reduction, and the plans must make recommendations to reduce structure ignitability. Plans are typically approved by a set of officials representing the local government, fire department, and state department of forestry or natural resources. Communities may be incentivized to develop a CWPP by virtue of being able to direct federal agency funds and actions toward community priorities. For example, to be eligible for federal funding to implement risk mitigation projects through Community Wildfire Defense Grants, communities needed to have a CWPP less than 10 years old. CWPPs also provide eligibility for state-level programs such as Tennessee’s Hazard Mitigation Assistance Grants and Community Education Grants. Thus, in addition to guiding the development of priorities for risk mitigation, CWPPs also help communities secure resources needed to implement risk mitigation.

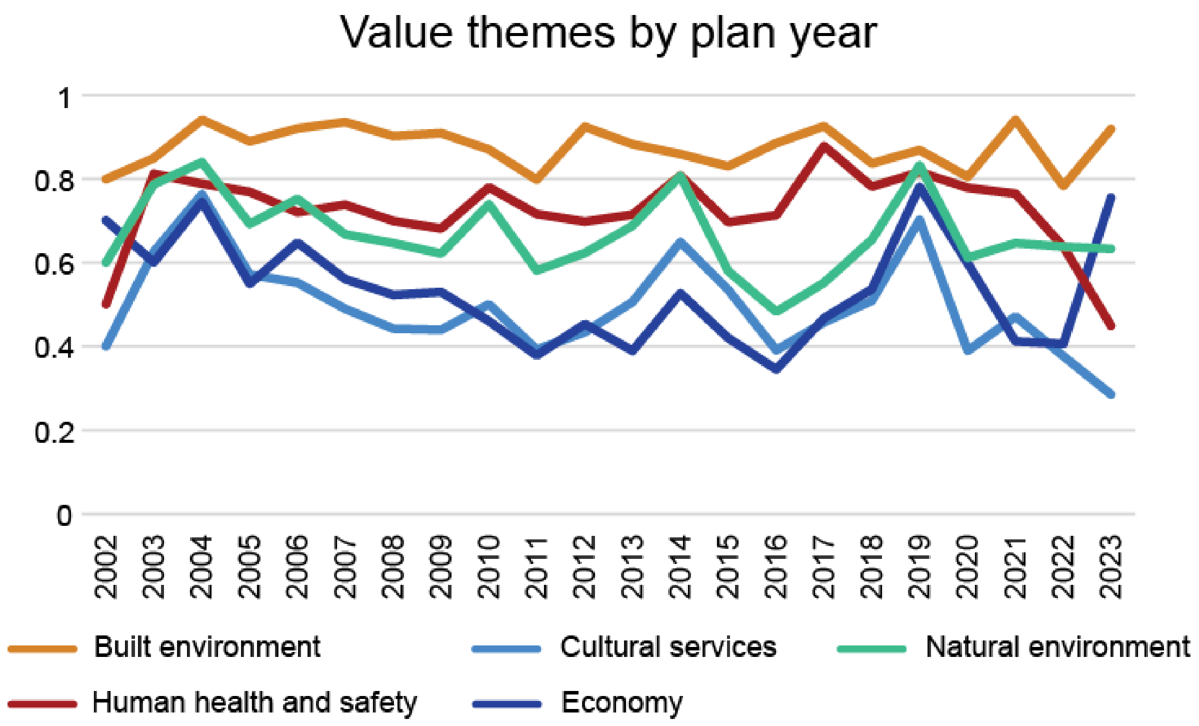

Fig. S1. Proportion of plans discussing each value theme over time.

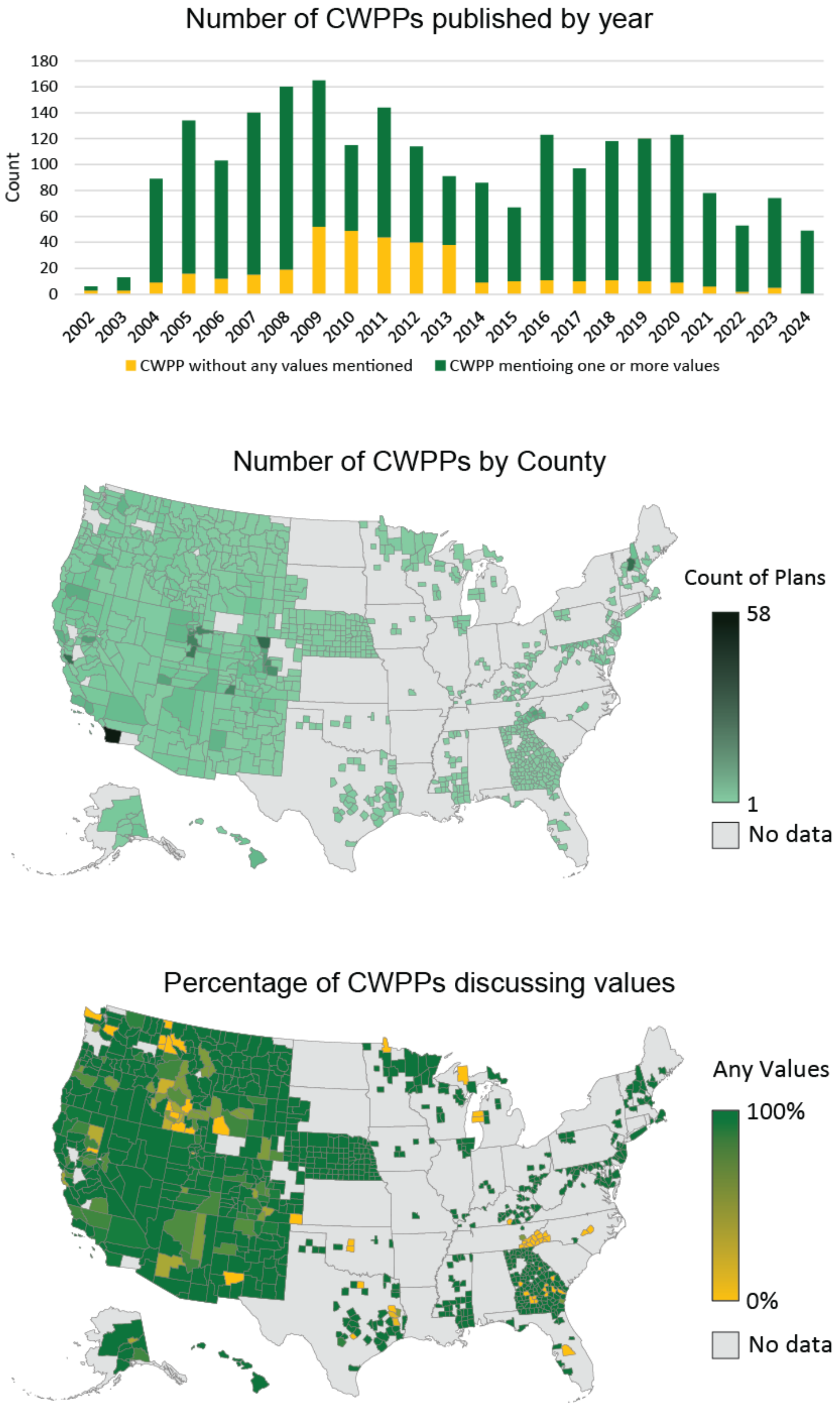

Fig. S2. Top: number of CWPPs published by year; green indicates plans containing values text. Middle: number of CWPPs by county. Bottom: percent of CWPPs containing values text by county.

Table S1. List of values

Value	Keywords	Frequency
**Built environment**		
cabin	cabin, cabins	5.6%
church	church, churches	7.1%
deck	deck, decks	3.8%
housing	condominium, condo, condos, duplex, dwelling, dwellings, home, homes, house, houses, housing, residence, residences	55.5%
infrastructure	infrastructural, infrastructure, infrastructures	54.7%
irrigation	irrigated, irrigating, irrigation	4.6%
railroad	railroad, railroads, railway, railways, trains	6.5%
reservoir	reservoir, reservoirs	9.0%
road	road, roads, highway, highways, street, streets, roadway, freeway, freeways, roadways	33.4%
school	school, schoolhouse, schoolhouses, schooling, schools	19.1%
structure	structure, structures	50.0%
**Cultural services**		
aesthetic	aesthetic, aesthetical, aesthetically, aesthetics, esthetic, esthetics, viewshed	8.9%
archaeology	archaeological, archaeologically, archaeology, archeological, historic site	27.0%
camp	camp, camped, camping, camps, tent, tents	11.0%
recreation	recreation, recreational, recreators, recreating, hiking, mountain biking	40.8%
**Economy**		
agriculture	agribusiness, agricultural, agriculturally, agriculture, farm, farmed, farming, farms	19.9%
industry	factories, factory, mill, milling, mills, industrial, industries, industry, manufacture, manufactured, manufacturer, manufacturers, manufacturing, commercial	29.2%
job	job, jobs, economy, worker, workers, employee, employees	16.8%
livestock	livestock, cattle, cow, cows	11.1%
logging	log, logged, logging, logs, lumber, timber	15.4%

Value	Keywords	Frequency
rangeland	rangeland, rangelands, pasture, pastures, forage, foraging	9.5%
ski	ski, skiing, skis	4.4%
taxes	taxes	0.4%
tourism	tourism, tourist, tourists, visitor, visitors	24.8%
**Health & human safety**		
drinking water	drinking water	42.2%
firefighter	firefight, firefighter, firefighters, fireman, firemen, firemans	12.8%
hospital	hospital, hospitalization, hospitalizations, hospitalized, hospitals	6.7%
medical	medical, medically, medications, medicine, medicines	4.3%
resident	resident, residents, household, households, homeowner	30.7%
safety	safety, lives	40.8%
**Natural environment**		
beach	beach, beaches	
bird	bird, birding, birds, bluebird, chicken, crane, creeper, cuckoo, dipper, dove, duck, eagle, emu, falcon, finch, flycatcher, fowl, gallina, goose, goshawk, grouse, harrier, hawk, hen, heron, hummingbird, ibis, kite, lark, lory, macaw, nighthawk, owl	14.8%
eagle	eagle, eagles	5.0%
owl	owl, owls	4.4%
coast	coast, coastal	4.3%
deer	deer, elk, elks, moose, wapiti	10.6%
ecosystem	ecosystem, ecosystems, ecologic, ecological, ecologically, ecology	22.3%
fish	fish, fishes, fishing, fishery, fisheries, salmon, salmonid, coho, chinook, trout, minnow	17.9%
salmon	salmon, salmonid, coho, chinook	2.9%
trout	trout	4.7%
grassland	grassland, grasslands, lea, meadow, meadows, prairie, prairies	11.8%
habitat	habitat, habitats	31.2%
lake	lake, lakes, lagoon, bayou, pond, ponds	19.1%
listed species	endangered species, threatened species, listed species, esa-listed	30.6%

**Value**	**Keywords**	**Frequency**
plants	flora, floras, plant, plants	18.8%
riparian	riparian	8.6%
river	river, rivers, creek, creeks, brook, brooks, stream, streams	31.4%
sagebrush	sagebrush	3.5%
species	species	23.7%
tree	tree, trees	26.0%
aspen	aspen, aspens	3.9%
cottonwood	cottonwood, cottonwoods	2.9%
fir	fir, firs	2.4%
juniper	juniper, junipers	3.3%
oak	oak, oaks	7.1%
pine	pine, pines	12.8%
ponderosa	ponderosa, ponderosas	5.2%
willow	willow, willows	3.0%
wilderness	wilderness, wildernesses	5.2%
wildlife	wildlife	38.8%
**Other**		
city	cities, city, metropolis, municipalities, municipality, town, towns, township, townships, hamlet, settlement, settlements, village, villages	29.4%
landowner	landowner, landowners, property owner, property owners	50.0%
tribe	tribe, tribal, tribally, amerind, native american, alaska native, american indian	6.2%
wui	wui, wuis, wildland urban interface, wildland-urban interface	47.7%

Table S2. Factors predicting discussion of *Built Environment* in Community Wildfire Protection Plans

	Model 1: No State			Model 2: State Fixed Effects		
**Parameter**	**Median**	**95% CI**	**pd**	**Median**	**95% CI**	**pd**
(Intercept)	2.29	(-0.08, 3.83)	97.25%	2.39	(0.42, 3.77)	98.43%
fire_risk	2.07e-03	(-0.07, 0.11)	54.90%	1.96e-03	(-0.06, 0.10)	58.12%
fire_intense	-8.90e-03	(-0.18, 0.09)	60.52%	4.38e-03	(-0.07, 0.13)	62.50%
Exposure	5.53e-03	(-0.09, 0.23)	58.83%	-1.70e-03	(-0.05, 0.03)	61.90%
WHP	-2.30e-04	(-0.14, 0.10)	51.08%	-2.92e-03	(-0.19, 0.06)	61.32%
ALR_VALA	0.01	(-0.02, 0.25)	77.90%	3.59e-03	(-0.02, 0.15)	69.22%
ALR_VALB	3.75e-03	(-0.07, 0.08)	61.08%	1.63e-03	(-0.04, 0.08)	59.00%
ALR_VALP	4.50e-03	(-0.02, 0.29)	67.10%	-1.06e-05	(-0.02, 0.08)	50.42%
PM25	-4.98e-03	(-0.17, 0.10)	58.55%	6.95e-04	(-0.07, 0.10)	53.92%
income	0.04	(-0.10, 0.37)	70.35%	-3.03e-04	(-0.10, 0.19)	51.48%
unemployed	2.12e-04	(-0.06, 0.08)	51.40%	3.11e-03	(-0.02, 0.11)	65.82%
poverty	7.13e-03	(-0.07, 0.23)	61.20%	5.99e-03	(-0.05, 0.15)	65.82%
asthma	0.01	(-0.06, 0.15)	67.92%	-1.16e-04	(-0.10, 0.10)	50.83%
diabetes	-0.01	(-0.41, 0.07)	63.70%	-3.05e-04	(-0.42, 0.09)	51.32%
expect	3.30e-03	(-0.06, 0.09)	61.75%	1.75e-03	(-0.04, 0.07)	61.50%
houseburden	1.85e-06	(-0.09, 0.05)	50.00%	-4.41e-04	(-0.08, 0.03)	55.88%
plumbing	4.15e-03	(-0.05, 0.09)	63.00%	3.33e-03	(-0.02, 0.09)	66.80%
transport	-2.98e-03	(-0.07, 0.07)	61.72%	-2.50e-03	(-0.08, 0.03)	65.97%
civic	-2.28e-03	(-0.11, 0.11)	55.30%	2.38e-03	(-0.08, 0.13)	59.87%
religious	-7.54e-04	(-0.10, 0.10)	53.17%	-1.62e-03	(-0.07, 0.06)	60.00%
fraternal	-6.06e-03	(-0.14, 0.04)	65.12%	-3.47e-03	(-0.12, 0.02)	66.55%
union	-0.02	(-0.30, 0.02)	79.97%	-1.12e-03	(-0.10, 0.03)	60.10%
charitable	0.01	(-0.03, 0.31)	70.47%	2.82e-03	(-0.04, 0.11)	63.77%
phone	-4.52e-05	(-0.05, 0.09)	50.28%	-9.42e-04	(-0.06, 0.12)	58.92%
language	0.01	(-0.06, 0.29)	69.70%	5.94e-03	(-0.03, 0.41)	68.60%
federal	4.65e-04	(-0.04, 0.07)	53.80%	-4.84e-05	(-0.04, 0.03)	51.18%
state	-2.35e-03	(-0.11, 0.04)	60.68%	1.01e-04	(-0.06, 0.04)	51.57%
local	-2.34e-03	(-0.12, 0.04)	60.75%	-3.65e-05	(-0.04, 0.05)	50.97%
educ_eq	-2.17e-04	(-0.16, 0.04)	51.95%	-9.18e-05	(-0.04, 0.04)	52.38%
employ	1.00e-03	(-0.06, 0.13)	55.73%	-5.29e-04	(-0.08, 0.04)	56.15%
ethnic_eq	-3.73e-03	(-0.24, 0.04)	65.10%	4.29e-05	(-0.06, 0.05)	50.77%
gender_inc	7.50e-04	(-0.05, 0.06)	58.05%	6.70e-05	(-0.02, 0.06)	52.52%
income_eq	-3.66e-04	(-0.16, 0.05)	53.00%	5.33e-05	(-0.04, 0.04)	51.40%
under65	-2.85e-04	(-0.17, 0.04)	53.62%	-2.38e-04	(-0.07, 0.03)	55.88%
civic_ops	-3.51e-05	(-0.05, 0.06)	50.55%	3.06e-04	(-0.02, 0.09)	57.38%
politic_act	-3.50e-04	(-0.12, 0.03)	55.33%	-5.05e-05	(-0.04, 0.04)	52.02%
over18	5.77e-04	(-0.05, 0.16)	57.38%	-1.52e-04	(-0.13, 0.03)	55.30%
**GROUP EFFECTS**						
Level.CNTY	0.01	(-1.53, 2.36)	51.15%	-0.05	(-1.30, 1.84)	55.63%
Level.COMM	-0.48	(-2.03, 1.88)	76.17%	-0.16	(-1.41, 1.72)	67.32%
Level.FPRD	1.02	(-0.44, 3.69)	93.12%	0.48	(-0.51, 2.83)	86.82%
State.AZ				0.27	(-0.94, 1.63)	65.10%
State.CA				-0.15	(-0.94, 0.61)	65.37%
State.CO				0.26	(-0.55, 1.05)	74.28%
State.DE				0.87	(-1.43, 3.86)	75.05%
State.FL				-1.24	(-2.59, 0.18)	96.22%
State.GA				2.13	(0.80, 3.96)	99.93%
State.IA				0.77	(-1.54, 4.02)	72.58%
State.ID				-0.14	(-1.22, 0.91)	60.12%
State.IL				0.98	(-1.20, 3.97)	78.83%
State.IN				-3.33	(-5.89, -1.34)	100%
State.KY				0.91	(-0.36, 2.47)	91.75%

	Model 1: No State			Model 2: State Fixed Effects		
**Parameter**	**Median**	**95% CI**	**pd**	**Median**	**95% CI**	**pd**
State.MA				0.90	(-1.33, 4.03)	76.47%
State.MD				2.10	(0.32, 4.89)	99.07%
State.ME				-1.30	(-3.10, 0.59)	91.25%
State.MI				-1.79	(-3.46, -0.12)	98.20%
State.MN				1.09	(-1.01, 4.08)	83.05%
State.MO				-1.98	(-5.17, 0.86)	91.45%
State.MS				-0.58	(-1.72, 0.67)	82.57%
State.MT				0.64	(-0.55, 2.02)	84.60%
State.NE				-1.74	(-3.02, -0.50)	99.68%
State.NH				2.36	(0.68, 4.97)	99.90%
State.NJ				-3.45	(-4.58, -2.42)	100%
State.NM				0.90	(-0.28, 2.31)	92.95%
State.NV				0.25	(-1.00, 1.67)	64.87%
State.NY				-0.53	(-2.54, 1.73)	69.20%
State.OH				0.20	(-1.53, 2.35)	58.42%
State.OK				0.69	(-1.70, 3.85)	70.13%
State.OR				0.25	(-0.72, 1.28)	68.77%
State.PA				-2.45	(-4.55, -0.59)	99.50%
State.RI				0.31	(-2.51, 3.65)	58.13%
State.SD				1.07	(-1.01, 4.14)	82.62%
State.TN				0.58	(-2.03, 3.89)	66.22%
State.TX				-0.71	(-1.61, 0.21)	93.80%
State.UT				-1.01	(-1.79, -0.29)	99.58%
State.VA				0.35	(-2.46, 3.73)	59.23%
State.VT				0.69	(-1.77, 3.88)	70.05%
State.WA				0.31	(-0.73, 1.45)	71.57%
State.WI				-0.20	(-1.46, 1.23)	61.48%
State.WV				1.40	(-0.53, 4.47)	90.88%
State.WY				-0.66	(-1.67, 0.44)	88.55%
Year.2002	0.01	(-0.50, 0.68)	55.17%	8.00e-03	(-0.43, 0.55)	54.35%
Year.2003	-0.03	(-0.66, 0.42)	60.27%	-0.01	(-0.54, 0.40)	55.25%
Year.2004	-0.08	(-0.58, 0.27)	70.30%	-0.03	(-0.46, 0.28)	61.70%
Year.2005	0.16	(-0.15, 0.73)	82.00%	0.07	(-0.20, 0.63)	71.15%
Year.2006	2.92e-03	(-0.41, 0.43)	51.15%	-0.03	(-0.49, 0.29)	61.77%
Year.2007	0.09	(-0.25, 0.60)	71.60%	0.03	(-0.26, 0.47)	61.05%
Year.2008	0.17	(-0.14, 0.72)	82.60%	0.06	(-0.20, 0.62)	71.47%
Year.2009	0.04	(-0.36, 0.52)	61.15%	2.63e-03	(-0.33, 0.40)	51.77%
Year.2010	0.04	(-0.33, 0.59)	62.15%	0.01	(-0.35, 0.47)	55.73%
Year.2011	-0.02	(-0.45, 0.35)	57.10%	0.01	(-0.34, 0.46)	55.92%
Year.2012	-0.18	(-0.77, 0.14)	83.08%	-0.05	(-0.57, 0.25)	66.28%
Year.2013	0.06	(-0.34, 0.65)	64.70%	5.33e-03	(-0.38, 0.45)	53.30%
Year.2014	9.11e-03	(-0.40, 0.47)	53.12%	0.03	(-0.30, 0.54)	61.67%
Year.2015	-0.07	(-0.64, 0.30)	67.22%	-0.03	(-0.54, 0.30)	61.47%
Year.2016	-0.01	(-0.43, 0.34)	54.85%	-0.02	(-0.45, 0.30)	59.98%
Year.2017	-1.46e-03	(-0.43, 0.42)	50.58%	-0.03	(-0.52, 0.31)	60.52%
Year.2018	0.09	(-0.22, 0.62)	71.47%	0.06	(-0.22, 0.59)	68.67%
Year.2019	-0.15	(-0.65, 0.17)	81.33%	-0.02	(-0.44, 0.29)	60.17%
Year.2020	0.03	(-0.33, 0.45)	58.83%	0.08	(-0.16, 0.60)	73.93%
Year.2021	-0.13	(-0.70, 0.20)	77.83%	-0.03	(-0.48, 0.27)	61.78%
Year.2022	0.09	(-0.30, 0.73)	69.03%	0.04	(-0.26, 0.65)	65.93%
Year.2023	-0.24	(-0.85, 0.11)	86.67%	-0.13	(-0.80, 0.13)	80.35%
Year.2024	0.07	(-0.31, 0.66)	65.45%	-0.02	(-0.57, 0.33)	59.78%

Table S3. Factors predicting discussion of *Cultural Services* in Community Wildfire Protection Plans

	Model 1: No State			Model 2: State Fixed Effects		
**Parameter**	**Median**	**95% CI**	**pd**	**Median**	**95% CI**	**pd**
(Intercept)	0.04	(-1.25, 1.30)	53.85%	-0.94	(-2.49, 0.79)	89.35%
fire_risk	-0.07	(-0.24, 0.11)	76.05%	2.56e-03	(-0.06, 0.07)	59.57%
fire_intense	0.29	(0.07, 0.52)	99.60%	8.74e-04	(-0.11, 0.07)	54.00%
Exposure	-0.20	(-0.33, -0.06)	99.70%	-3.49e-05	(-0.05, 0.03)	50.50%
WHP	-0.11	(-0.30, 0.10)	84.15%	-3.10e-03	(-0.09, 0.07)	60.97%
ALR_VALA	0.09	(-0.03, 0.20)	92.85%	3.11e-03	(-0.02, 0.04)	67.30%
ALR_VALB	0.12	(-0.02, 0.27)	95.88%	4.42e-04	(-0.03, 0.05)	53.65%
ALR_VALP	0.02	(-0.04, 0.14)	69.35%	1.74e-03	(-0.01, 0.14)	65.88%
PM25	-0.65	(-0.85, -0.45)	100%	1.46e-03	(-0.09, 0.06)	55.40%
income	0.10	(-0.13, 0.36)	79.92%	0.01	(-0.05, 0.16)	69.13%
unemployed	0.07	(-0.07, 0.22)	83.70%	2.17e-03	(-0.02, 0.06)	62.75%
poverty	-0.18	(-0.41, 0.03)	94.92%	4.59e-04	(-0.08, 0.06)	52.33%
asthma	0.13	(-0.03, 0.33)	92.80%	-8.09e-03	(-0.09, 0.04)	69.68%
diabetes	-0.05	(-0.34, 0.17)	63.50%	6.67e-03	(-0.05, 0.13)	66.63%
expect	-2.87e-03	(-0.11, 0.16)	51.48%	2.29e-03	(-0.05, 0.04)	62.95%
houseburden	-0.09	(-0.20, -0.00)	97.80%	-5.03e-03	(-0.05, 0.02)	72.83%
plumbing	0.03	(-0.08, 0.18)	68.80%	4.92e-03	(-0.02, 0.06)	69.45%
transport	0.05	(-0.04, 0.20)	84.92%	-6.46e-03	(-0.07, 0.02)	72.80%
civic	0.12	(-0.09, 0.31)	87.52%	7.66e-03	(-0.04, 0.12)	65.23%
religious	0.07	(-0.10, 0.19)	81.88%	2.54e-03	(-0.05, 0.06)	62.27%
fraternal	-0.02	(-0.14, 0.10)	65.92%	-0.01	(-0.10, 0.01)	78.25%
union	-0.11	(-0.25, 0.00)	96.80%	-9.02e-03	(-0.12, 0.02)	75.42%
charitable	0.15	(-0.01, 0.35)	95.70%	0.02	(-0.02, 0.20)	77.55%
phone	0.13	(-0.02, 0.29)	94.47%	-6.46e-03	(-0.07, 0.03)	72.42%
language	-0.24	(-0.45, -0.04)	98.72%	6.98e-04	(-0.05, 0.17)	54.33%
federal	-0.02	(-0.10, 0.10)	63.15%	-3.95e-03	(-0.06, 0.02)	70.33%
state	-0.07	(-0.21, 0.02)	94.17%	-4.67e-04	(-0.12, 0.02)	55.27%
local	-7.28e-03	(-0.10, 0.12)	55.33%	-8.79e-04	(-0.06, 0.03)	57.75%
educ_eq	0.25	(0.10, 0.40)	99.98%	-2.04e-03	(-0.04, 0.03)	64.48%
employ_eq	-0.25	(-0.46, -0.05)	99.70%	-4.59e-03	(-0.10, 0.03)	68.93%
ethnic_eq	0.03	(-0.09, 0.21)	68.60%	-2.62e-03	(-0.07, 0.04)	64.38%
gender_inc	-0.06	(-0.19, 0.00)	96.15%	-3.12e-03	(-0.06, 0.02)	69.97%
income_eq	0.22	(0.08, 0.37)	99.88%	9.95e-03	(-0.01, 0.31)	74.17%
under65	0.01	(-0.06, 0.15)	61.92%	4.78e-04	(-0.02, 0.08)	58.57%
civic_ops	0.02	(-0.07, 0.12)	69.40%	4.14e-05	(-0.03, 0.04)	51.42%
politic_act	-0.02	(-0.11, 0.07)	69.90%	-4.38e-05	(-0.04, 0.03)	51.62%
over18	-0.10	(-0.32, 0.05)	87.10%	5.28e-05	(-0.04, 0.06)	52.57%
**GROUP EFFECTS**						
Level.CNTY	0.09	(-1.12, 1.37)	61.20%	0.11	(-1.50, 1.35)	60.18%
Level.COMM	-0.45	(-1.70, 0.82)	84.47%	-0.57	(-2.19, 0.63)	88.87%
Level.FPRD	0.38	(-0.84, 1.70)	81.88%	0.32	(-1.25, 1.63)	75.80%
State.AZ				2.19	(1.01, 3.45)	99.98%
State.CA				2.09	(1.17, 3.14)	100%
State.CO				1.64	(0.72, 2.69)	99.92%
State.DE				-1.87	(-6.38, 1.07)	87.92%
State.FL				-1.55	(-4.00, 0.31)	94.83%
State.GA				-5.19	(-8.64, -3.19)	100%
State.IA				2.07	(-0.13, 4.66)	96.82%
State.ID				1.07	(-0.01, 2.25)	97.38%
State.IL				2.94	(1.05, 5.40)	99.82%
State.IN				-2.70	(-6.94, -0.00)	97.52%
State.KY				-4.30	(-7.99, -2.17)	100%

	Model 1: No State			Model 2: State Fixed Effects		
**Parameter**	**Median**	**95% CI**	**pd**	**Median**	**95% CI**	**pd**
State.MA				1.73	(-0.28, 3.81)	95.13%
State.MD				0.91	(-0.16, 2.05)	95.48%
State.ME				-2.19	(-6.53, 0.56)	93.43%
State.MI				1.00	(-0.88, 2.98)	86.15%
State.MN				1.79	(0.15, 3.78)	98.30%
State.MO				-1.42	(-6.38, 2.22)	77.95%
State.MS				-2.55	(-4.92, -0.81)	99.90%
State.MT				1.59	(0.52, 2.76)	99.78%
State.NE				-2.58	(-6.85, 0.00)	97.42%
State.NH				1.07	(-0.01, 2.19)	97.23%
State.NJ				-3.35	(-7.21, -1.07)	99.93%
State.NM				2.10	(1.06, 3.28)	99.98%
State.NV				2.96	(1.74, 4.40)	100%
State.NY				1.33	(-0.92, 3.56)	88.07%
State.OH				-0.65	(-2.63, 0.98)	77.70%
State.OK				-1.70	(-6.24, 1.18)	85.72%
State.OR				1.37	(0.40, 2.47)	99.68%
State.PA				-2.55	(-6.77, 0.19)	96.35%
State.RI				-1.20	(-6.14, 2.46)	73.92%
State.SD				4.16	(1.82, 7.92)	99.98%
State.TN				-1.08	(-5.94, 2.24)	73.12%
State.TX				0.16	(-0.91, 1.29)	61.78%
State.UT				2.79	(1.82, 3.88)	100%
State.VA				-1.01	(-6.03, 2.76)	68.60%
State.VT				1.89	(-0.56, 4.72)	93.20%
State.WA				0.38	(-0.67, 1.48)	76.83%
State.WI				1.80	(0.55, 3.12)	99.85%
State.WV				-2.83	(-6.85, -0.32)	98.82%
State.WY				0.38	(-0.73, 1.59)	75.13%
Year.2002	-0.07	(-0.95, 0.76)	56.03%	-0.23	(-1.30, 0.72)	68.22%
Year.2003	-0.26	(-1.07, 0.44)	77.25%	-0.22	(-1.15, 0.62)	71.37%
Year.2004	-0.07	(-0.54, 0.38)	62.05%	-0.08	(-0.59, 0.44)	61.08%
Year.2005	0.74	(0.32, 1.22)	99.92%	0.69	(0.18, 1.22)	99.63%
Year.2006	0.04	(-0.40, 0.47)	57.88%	0.02	(-0.48, 0.51)	53.58%
Year.2007	0.11	(-0.29, 0.51)	69.90%	0.12	(-0.34, 0.60)	69.25%
Year.2008	0.07	(-0.32, 0.47)	65.22%	0.15	(-0.33, 0.63)	73.27%
Year.2009	-0.26	(-0.68, 0.14)	89.98%	-0.36	(-0.83, 0.11)	93.40%
Year.2010	-0.24	(-0.73, 0.21)	85.58%	-0.51	(-1.08, 0.01)	97.07%
Year.2011	0.01	(-0.41, 0.44)	52.52%	0.15	(-0.33, 0.66)	72.23%
Year.2012	-0.17	(-0.64, 0.30)	76.65%	0.05	(-0.52, 0.64)	57.38%
Year.2013	-0.04	(-0.56, 0.47)	56.40%	-0.16	(-0.76, 0.44)	70.20%
Year.2014	-0.04	(-0.49, 0.40)	56.88%	0.17	(-0.34, 0.71)	74.73%
Year.2015	0.30	(-0.20, 0.84)	88.83%	0.39	(-0.18, 1.01)	91.37%
Year.2016	0.30	(-0.13, 0.76)	92.38%	0.24	(-0.24, 0.75)	83.17%
Year.2017	0.24	(-0.21, 0.72)	85.60%	0.72	(0.13, 1.39)	99.22%
Year.2018	0.23	(-0.19, 0.67)	85.65%	0.62	(0.07, 1.24)	98.68%
Year.2019	0.15	(-0.26, 0.59)	75.75%	0.24	(-0.26, 0.79)	83.03%
Year.2020	0.60	(0.17, 1.09)	99.55%	0.41	(-0.11, 0.96)	94.22%
Year.2021	-0.47	(-0.93, -0.00)	97.70%	-0.81	(-1.40, -0.28)	99.85%
Year.2022	-0.12	(-0.65, 0.37)	67.12%	-0.28	(-0.88, 0.30)	82.67%
Year.2023	-0.50	(-1.00, -0.04)	98.20%	-0.67	(-1.22, -0.15)	99.52%
Year.2024	-0.55	(-1.13, -0.04)	98.25%	-0.68	(-1.35, -0.09)	98.92%

Table S4. Factors predicting discussion of *Economy* in Community Wildfire Protection Plans

	Model 1: No State			Model 2: State Fixed Effects		
**Parameter**	**Median**	**95% CI**	**pd**	**Median**	**95% CI**	**pd**
(Intercept)	0.05	(-1.02, 1.18)	55.73%	-0.44	(-1.70, 0.88)	79.63%
fire_risk	-0.06	(-0.24, 0.12)	74.25%	-8.05e-03	(-0.13, 0.04)	68.63%
fire_intense	0.12	(-0.10, 0.34)	85.70%	-1.87e-03	(-0.19, 0.09)	55.37%
Exposure	-0.07	(-0.16, 0.03)	93.35%	-3.43e-03	(-0.08, 0.11)	63.62%
WHP	-0.06	(-0.27, 0.15)	71.40%	6.67e-03	(-0.05, 0.13)	66.03%
ALR_VALA	0.05	(-0.05, 0.17)	78.33%	3.66e-03	(-0.02, 0.12)	66.75%
ALR_VALB	0.07	(-0.05, 0.17)	89.92%	4.48e-04	(-0.09, 0.06)	53.12%
ALR_VALP	0.03	(-0.04, 0.16)	75.55%	1.54e-03	(-0.02, 0.08)	62.33%
PM25	-0.54	(-0.73, -0.34)	100%	-4.78e-03	(-0.22, 0.05)	61.48%
income	0.10	(-0.14, 0.33)	80.65%	7.85e-03	(-0.08, 0.25)	62.83%
unemployed	-9.67e-03	(-0.11, 0.14)	56.20%	1.26e-03	(-0.03, 0.14)	58.10%
poverty	-0.24	(-0.47, -0.02)	98.47%	-4.39e-03	(-0.16, 0.05)	61.30%
asthma	0.40	(0.21, 0.59)	100%	6.92e-03	(-0.03, 0.28)	69.00%
diabetes	-0.09	(-0.37, 0.13)	75.88%	-1.27e-03	(-0.09, 0.14)	54.87%
expect	0.14	(-0.03, 0.31)	94.38%	-3.75e-04	(-0.05, 0.05)	52.90%
houseburden	-0.18	(-0.31, -0.04)	99.42%	-4.27e-03	(-0.16, 0.02)	70.68%
plumbing	0.02	(-0.07, 0.14)	70.20%	8.68e-04	(-0.04, 0.06)	56.88%
transport	0.07	(-0.03, 0.19)	92.85%	-5.40e-05	(-0.06, 0.04)	50.77%
civic	-0.07	(-0.22, 0.08)	82.30%	-3.33e-03	(-0.09, 0.05)	61.23%
religious	0.07	(-0.05, 0.22)	88.78%	2.33e-03	(-0.03, 0.10)	62.47%
fraternal	0.01	(-0.10, 0.10)	60.20%	-2.85e-03	(-0.08, 0.03)	63.92%
union	-0.08	(-0.22, 0.03)	93.62%	-7.07e-04	(-0.06, 0.05)	55.80%
charitable	0.04	(-0.10, 0.17)	76.42%	1.94e-03	(-0.05, 0.09)	59.47%
phone	0.26	(0.09, 0.41)	99.92%	2.30e-03	(-0.02, 0.21)	65.08%
language	-0.27	(-0.49, -0.04)	98.83%	-1.25e-04	(-0.06, 0.10)	50.93%
federal	-0.01	(-0.13, 0.06)	61.98%	-1.05e-04	(-0.05, 0.03)	51.92%
state	-5.02e-03	(-0.11, 0.08)	55.38%	-1.06e-03	(-0.05, 0.04)	59.52%
local	0.04	(-0.05, 0.17)	78.72%	3.63e-04	(-0.04, 0.07)	54.08%
educ_eq	0.24	(0.08, 0.38)	99.85%	1.11e-03	(-0.02, 0.10)	62.48%
employ_eq	-0.07	(-0.28, 0.05)	86.48%	3.06e-04	(-0.06, 0.06)	53.37%
ethnic_eq	0.19	(4.89e-03, 0.40)	98.00%	-1.02e-04	(-0.14, 0.05)	51.58%
gender_inc	-0.02	(-0.12, 0.06)	72.22%	5.47e-05	(-0.04, 0.03)	51.80%
income_eq	-0.03	(-0.12, 0.08)	75.02%	-2.46e-04	(-0.05, 0.03)	54.27%
under65	0.03	(-0.06, 0.18)	69.23%	1.16e-03	(-0.02, 0.15)	63.05%
civic_ops	-0.03	(-0.19, 0.05)	72.90%	1.68e-04	(-0.03, 0.05)	53.97%
politic_act	-0.02	(-0.13, 0.05)	77.18%	-3.37e-04	(-0.08, 0.02)	56.60%
over18	-0.13	(-0.35, 0.02)	92.07%	2.04e-05	(-0.06, 0.06)	50.98%
**GROUP EFFECTS**						
Level.CNTY	0.37	(-0.73, 1.50)	85.20%	0.26	(-0.91, 1.29)	77.58%
Level.COMM	-0.17	(-1.29, 0.90)	69.33%	-0.37	(-1.62, 0.57)	86.43%
Level.FPRD	-0.21	(-1.34, 0.81)	73.60%	0.04	(-1.17, 1.03)	54.52%
State.AZ				0.88	(-0.15, 1.90)	95.33%
State.CA				0.91	(0.15, 1.77)	99.10%
State.CO				0.88	(0.12, 1.74)	98.83%
State.DE				-1.91	(-5.64, 0.66)	91.60%
State.FL				-0.81	(-2.36, 0.61)	86.62%
State.GA				-3.89	(-5.52, -2.60)	100%
State.IA				1.48	(-0.56, 3.95)	92.03%
State.ID				1.75	(0.74, 2.89)	99.97%
State.IL				1.17	(-0.57, 3.17)	90.50%
State.IN				-2.32	(-5.88, 0.14)	96.83%
State.KY				-1.50	(-2.61, -0.44)	99.75%

	Model 1: No State			Model 2: State Fixed Effects		
**Parameter**	**Median**	**95% CI**	**pd**	**Median**	**95% CI**	**pd**
State.MA				1.72	(-0.22, 4.17)	95.77%
State.MD				-4.15	(-7.04, -2.21)	100%
State.ME				-2.13	(-5.72, 0.29)	95.70%
State.MI				-0.31	(-2.25, 1.37)	63.47%
State.MN				0.93	(-0.55, 2.53)	88.73%
State.MO				-1.18	(-5.18, 2.01)	75.53%
State.MS				-1.84	(-3.10, -0.64)	99.80%
State.MT				2.36	(1.31, 3.57)	100%
State.NE				-0.37	(-2.02, 1.06)	69.45%
State.NH				5.26	(3.49, 8.04)	100%
State.NJ				-2.31	(-4.47, -0.74)	99.80%
State.NM				1.19	(0.27, 2.18)	99.42%
State.NV				2.07	(0.95, 3.30)	99.98%
State.NY				1.70	(-0.33, 4.08)	94.60%
State.OH				1.45	(0.02, 3.02)	97.62%
State.OK				-1.82	(-5.62, 0.80)	90.52%
State.OR				0.55	(-0.29, 1.47)	90.00%
State.PA				1.24	(-0.84, 3.59)	88.35%
State.RI				-1.22	(-5.23, 1.95)	76.68%
State.SD				2.04	(0.33, 4.34)	99.03%
State.TN				-1.37	(-5.27, 1.49)	81.50%
State.TX				1.06	(0.17, 2.04)	98.85%
State.UT				2.74	(1.91, 3.69)	100%
State.VA				-1.08	(-5.24, 2.26)	72.93%
State.VT				1.05	(-1.09, 3.49)	82.50%
State.WA				0.68	(-0.21, 1.64)	93.17%
State.WI				-0.41	(-1.72, 0.79)	73.73%
State.WV				-2.94	(-6.32, -0.78)	99.73%
State.WY				-0.31	(-1.30, 0.73)	71.80%
Year.2002	0.18	(-0.57, 1.10)	68.77%	0.10	(-0.60, 0.90)	62.72%
Year.2003	-9.50e-03	(-0.73, 0.72)	51.10%	-0.01	(-0.73, 0.69)	51.67%
Year.2004	-0.36	(-0.81, 0.10)	93.73%	-0.46	(-0.99, -0.01)	97.80%
Year.2005	0.44	(-0.00, 0.88)	97.45%	0.33	(-0.08, 0.80)	94.18%
Year.2006	-0.20	(-0.64, 0.21)	81.30%	-0.11	(-0.54, 0.31)	70.75%
Year.2007	0.36	(-0.05, 0.78)	95.58%	0.41	(0.01, 0.83)	97.70%
Year.2008	0.14	(-0.25, 0.53)	76.05%	0.38	(-0.03, 0.83)	96.48%
Year.2009	-0.03	(-0.44, 0.38)	57.07%	-0.01	(-0.42, 0.41)	52.40%
Year.2010	-0.07	(-0.57, 0.42)	60.85%	0.05	(-0.45, 0.56)	58.55%
Year.2011	-0.13	(-0.56, 0.27)	73.38%	0.03	(-0.40, 0.46)	55.07%
Year.2012	-0.30	(-0.81, 0.17)	89.75%	-0.03	(-0.52, 0.46)	55.53%
Year.2013	-0.05	(-0.57, 0.45)	58.20%	0.08	(-0.44, 0.63)	62.27%
Year.2014	-0.40	(-0.89, 0.06)	95.75%	-0.28	(-0.78, 0.18)	87.78%
Year.2015	-0.04	(-0.51, 0.45)	56.05%	0.07	(-0.41, 0.58)	61.12%
Year.2016	-0.16	(-0.59, 0.26)	76.02%	-0.14	(-0.58, 0.28)	75.32%
Year.2017	-0.17	(-0.63, 0.30)	76.02%	-0.02	(-0.55, 0.51)	52.73%
Year.2018	0.10	(-0.31, 0.52)	68.00%	0.37	(-0.10, 0.92)	94.02%
Year.2019	0.09	(-0.32, 0.51)	67.55%	0.07	(-0.35, 0.52)	62.45%
Year.2020	0.81	(0.34, 1.34)	100%	0.39	(-0.10, 0.92)	94.20%
Year.2021	0.11	(-0.34, 0.59)	68.88%	-0.35	(-0.89, 0.12)	92.93%
Year.2022	-0.38	(-0.90, 0.11)	93.80%	-0.35	(-0.91, 0.14)	91.35%
Year.2023	-0.49	(-0.98, -0.02)	98.08%	-0.46	(-0.96, -0.00)	97.55%
Year.2024	0.56	(0.04, 1.16)	98.38%	-0.04	(-0.61, 0.53)	55.30%

Table S5. Factors predicting discussion of *Human Health & Safety* in Community Wildfire Protection Plans

	Model 1: No State			Model 2: State Fixed Effects		
**Parameter**	**Median**	**95% CI**	**pd**	**Median**	**95% CI**	**pd**
(Intercept)	1.19	(0.09, 2.00)	97.67%	0.85	(0.04, 1.62)	97.77%
fire_risk	-0.27	(-0.43, -0.09)	99.85%	-0.21	(-0.38, -0.00)	97.73%
fire_intense	-0.04	(-0.26, 0.18)	63.92%	-0.16	(-0.36, 0.00)	96.48%
Exposure	0.14	(-0.01, 0.27)	96.03%	0.08	(-0.01, 0.19)	95.32%
WHP	0.48	(0.24, 0.73)	100%	0.37	(0.02, 0.64)	98.83%
ALR_VALA	0.18	(0.03, 0.35)	98.90%	0.08	(-0.02, 0.32)	88.92%
ALR_VALB	-0.16	(-0.30, -0.05)	99.92%	-0.03	(-0.20, 0.04)	74.18%
ALR_VALP	0.02	(-0.05, 0.25)	61.30%	-8.53e-03	(-0.06, 0.11)	65.73%
PM25	0.08	(-0.08, 0.26)	81.70%	0.01	(-0.17, 0.13)	60.25%
income	-0.11	(-0.33, 0.11)	83.55%	-0.02	(-0.27, 0.12)	62.02%
unemployed	0.20	(0.05, 0.35)	99.60%	0.03	(-0.04, 0.24)	72.50%
poverty	0.07	(-0.09, 0.25)	80.12%	2.63e-03	(-0.12, 0.12)	52.83%
asthma	-0.26	(-0.45, -0.07)	99.70%	-0.02	(-0.15, 0.07)	73.75%
diabetes	-0.07	(-0.25, 0.16)	76.55%	0.01	(-0.12, 0.33)	56.53%
expect	9.70e-03	(-0.09, 0.13)	58.98%	-9.75e-03	(-0.10, 0.06)	65.00%
houseburden	-7.31e-03	(-0.09, 0.09)	57.35%	-0.01	(-0.13, 0.04)	64.28%
plumbing	0.10	(-0.03, 0.26)	92.55%	-4.10e-03	(-0.07, 0.08)	57.13%
transport	-0.11	(-0.20, 0.04)	92.22%	-0.04	(-0.13, 0.06)	83.50%
civic	0.07	(-0.08, 0.25)	82.83%	2.53e-03	(-0.13, 0.12)	52.60%
religious	0.03	(-0.10, 0.16)	75.10%	5.77e-03	(-0.09, 0.11)	58.40%
fraternal	-0.05	(-0.15, 0.08)	80.38%	-0.03	(-0.12, 0.05)	85.87%
union	0.15	(-0.00, 0.29)	96.95%	0.06	(-0.03, 0.25)	83.02%
charitable	0.02	(-0.11, 0.18)	64.48%	5.17e-03	(-0.10, 0.12)	56.97%
phone	3.70e-03	(-0.10, 0.10)	54.52%	-5.80e-03	(-0.10, 0.06)	59.98%
language	0.22	(0.04, 0.40)	98.95%	-2.34e-03	(-0.14, 0.18)	52.12%
federal	0.34	(0.18, 0.52)	100%	0.18	(-0.02, 0.39)	91.18%
state	-0.04	(-0.18, 0.05)	79.12%	-0.01	(-0.12, 0.04)	67.97%
local	0.10	(-0.06, 0.27)	86.05%	-4.80e-03	(-0.11, 0.06)	59.38%
educ_eq	0.01	(-0.06, 0.15)	61.48%	0.02	(-0.03, 0.25)	69.97%
employ_eq	6.05e-03	(-0.11, 0.21)	53.67%	-2.29e-03	(-0.11, 0.09)	54.45%
ethnic_eq	-0.34	(-0.55, -0.12)	99.90%	3.45e-03	(-0.07, 0.18)	56.70%
gender_inc	-0.02	(-0.09, 0.09)	69.10%	-4.83e-03	(-0.07, 0.05)	64.83%
income_eq	0.19	(0.02, 0.34)	98.75%	0.04	(-0.03, 0.26)	79.33%
under65	3.54e-03	(-0.08, 0.12)	54.75%	7.50e-03	(-0.03, 0.15)	67.65%
civic_ops	0.07	(-0.02, 0.23)	91.00%	1.93e-03	(-0.05, 0.13)	56.52%
politic_act	8.71e-03	(-0.06, 0.14)	61.68%	5.68e-03	(-0.03, 0.14)	66.62%
over18	-0.03	(-0.23, 0.08)	72.47%	5.99e-04	(-0.10, 0.14)	53.35%
**GROUP EFFECTS**						
Level.CNTY	0.15	(-0.57, 1.29)	75.85%	-4.25e-03	(-0.58, 0.61)	53.45%
Level.COMM	-0.17	(-0.98, 0.89)	78.17%	-0.01	(-0.59, 0.56)	57.30%
Level.FPRD	0.07	(-0.66, 1.30)	62.92%	0.02	(-0.43, 0.79)	61.55%
State.AZ				0.75	(-0.25, 1.88)	93.15%
State.CA				0.56	(-0.14, 1.27)	94.12%
State.CO				0.74	(0.06, 1.40)	98.18%
State.DE				-2.12	(-4.56, -0.25)	98.88%
State.FL				-0.38	(-1.58, 0.87)	72.87%
State.GA				3.75	(2.31, 5.86)	100%
State.IA				1.48	(-0.42, 4.23)	93.27%
State.ID				0.21	(-0.66, 1.11)	68.43%
State.IL				0.02	(-1.63, 1.79)	51.05%
State.IN				-1.28	(-3.28, 0.54)	91.83%

	Model 1: No State			Model 2: State Fixed Effects		
**Parameter**	**Median**	**95% CI**	**pd**	**Median**	**95% CI**	**pd**
State.KY				-2.32	(-3.25, -1.46)	100%
State.MA				-0.18	(-1.87, 1.64)	58.27%
State.MD				3.05	(1.59, 5.25)	100%
State.ME				-0.35	(-2.06, 1.49)	65.83%
State.MI				-0.03	(-1.64, 1.71)	51.25%
State.MN				1.05	(-0.46, 3.05)	91.57%
State.MO				0.58	(-1.99, 3.56)	66.58%
State.MS				-0.40	(-1.38, 0.57)	80.37%
State.MT				0.80	(-0.06, 1.74)	96.65%
State.NE				-0.98	(-2.21, 0.23)	94.40%
State.NH				-1.15	(-1.99, -0.33)	99.73%
State.NJ				2.56	(0.96, 4.92)	99.97%
State.NM				0.50	(-0.37, 1.39)	86.57%
State.NV				0.29	(-0.79, 1.35)	70.47%
State.NY				0.18	(-1.67, 2.28)	57.32%
State.OH				0.71	(-0.62, 2.32)	83.40%
State.OK				-0.88	(-3.07, 1.04)	81.95%
State.OR				-0.19	(-0.93, 0.53)	70.25%
State.PA				-1.27	(-3.30, 0.47)	91.80%
State.RI				-1.22	(-4.13, 1.45)	82.63%
State.SD				0.33	(-1.18, 2.01)	65.87%
State.TN				-0.46	(-2.71, 1.85)	66.05%
State.TX				-0.13	(-0.93, 0.69)	62.93%
State.UT				0.33	(-0.40, 1.02)	81.35%
State.VA				-1.06	(-3.84, 1.44)	80.25%
State.VT				-0.05	(-1.94, 2.13)	52.08%
State.WA				0.76	(-0.07, 1.62)	96.28%
State.WI				-0.30	(-1.31, 0.73)	71.50%
State.WV				-3.39	(-5.61, -1.90)	100%
State.WY				-0.19	(-1.07, 0.70)	66.17%
Year.2002	-0.01	(-0.70, 0.67)	51.50%	-0.01	(-0.64, 0.53)	52.47%
Year.2003	-0.19	(-0.94, 0.38)	74.78%	-0.07	(-0.70, 0.39)	63.35%
Year.2004	0.29	(-0.12, 0.82)	90.77%	0.17	(-0.17, 0.69)	81.45%
Year.2005	0.15	(-0.23, 0.59)	77.05%	0.12	(-0.22, 0.54)	75.52%
Year.2006	0.07	(-0.34, 0.51)	63.50%	0.03	(-0.34, 0.45)	58.37%
Year.2007	5.26e-03	(-0.37, 0.42)	50.78%	0.03	(-0.32, 0.41)	57.62%
Year.2008	0.09	(-0.29, 0.47)	67.47%	0.14	(-0.17, 0.60)	80.62%
Year.2009	-0.13	(-0.53, 0.25)	76.10%	-0.01	(-0.41, 0.34)	54.25%
Year.2010	-0.22	(-0.71, 0.21)	83.40%	-0.17	(-0.70, 0.19)	81.28%
Year.2011	0.07	(-0.33, 0.49)	61.88%	0.02	(-0.37, 0.44)	54.62%
Year.2012	-0.13	(-0.57, 0.31)	71.75%	0.02	(-0.41, 0.49)	53.50%
Year.2013	-0.14	(-0.66, 0.32)	72.62%	-0.12	(-0.65, 0.27)	73.23%
Year.2014	-0.03	(-0.46, 0.41)	56.65%	-0.09	(-0.54, 0.29)	67.90%
Year.2015	0.10	(-0.35, 0.62)	67.33%	0.03	(-0.37, 0.50)	56.77%
Year.2016	-0.19	(-0.61, 0.22)	81.75%	-0.18	(-0.61, 0.15)	84.37%
Year.2017	-0.09	(-0.52, 0.34)	67.07%	-0.15	(-0.69, 0.22)	78.83%
Year.2018	0.50	(0.06, 1.03)	98.98%	0.21	(-0.14, 0.74)	85.57%
Year.2019	0.14	(-0.23, 0.60)	77.70%	0.02	(-0.33, 0.42)	56.87%
Year.2020	0.34	(-0.07, 0.83)	94.00%	0.28	(-0.07, 0.79)	91.98%
Year.2021	0.17	(-0.25, 0.67)	77.55%	0.19	(-0.17, 0.71)	83.47%
Year.2022	0.05	(-0.41, 0.55)	59.35%	0.05	(-0.34, 0.55)	61.95%
Year.2023	-0.32	(-0.83, 0.10)	93.40%	-0.24	(-0.76, 0.10)	89.03%
Year.2024	-0.52	(-1.10, -0.01)	97.95%	-0.22	(-0.81, 0.15)	84.90%

Table S6. Factors predicting discussion of *Natural Environment* in Community Wildfire Protection Plans

	**Model 1: No State**			**Model 2: State Fixed Effects**		
**Parameter**	**Median**	**95% CI**	**pd**	**Median**	**95% CI**	**pd**
(Intercept)	0.96	(-0.21, 1.88)	96.30%	0.46	(-1.03, 1.87)	78.82%
fire_risk	0.09	(-0.09, 0.28)	82.93%	-4.51e-03	(-0.09, 0.09)	58.70%
fire_intense	0.77	(0.52, 1.04)	100%	0.02	(-0.07, 0.19)	68.70%
Exposure	-0.25	(-0.39, -0.12)	100%	-0.02	(-0.15, 0.03)	79.65%
WHP	-0.25	(-0.48, -0.02)	98.15%	9.77e-04	(-0.13, 0.08)	52.63%
ALR_VALA	0.19	(0.05, 0.36)	99.52%	0.01	(-0.02, 0.15)	77.15%
ALR_VALB	-0.05	(-0.16, 0.07)	80.47%	-3.18e-04	(-0.17, 0.05)	51.17%
ALR_VALP	0.05	(-0.05, 0.29)	75.08%	-3.36e-03	(-0.04, 0.11)	62.93%
PM25	-0.16	(-0.38, 0.06)	92.83%	0.02	(-0.07, 0.19)	70.23%
income	0.15	(-0.06, 0.39)	91.77%	0.02	(-0.07, 0.26)	68.58%
unemployed	0.05	(-0.06, 0.20)	77.35%	5.47e-03	(-0.04, 0.08)	68.88%
poverty	-4.20e-03	(-0.24, 0.18)	52.08%	-2.08e-03	(-0.16, 0.08)	54.60%
asthma	0.07	(-0.10, 0.22)	81.90%	0.02	(-0.04, 0.17)	76.83%
diabetes	-0.14	(-0.42, 0.09)	87.67%	-0.02	(-0.17, 0.11)	69.00%
expect	0.17	(-0.02, 0.36)	95.83%	3.99e-03	(-0.03, 0.13)	62.70%
houseburden	-0.10	(-0.25, 0.02)	92.88%	-5.96e-03	(-0.10, 0.03)	67.62%
plumbing	-0.03	(-0.14, 0.09)	72.42%	-0.02	(-0.24, 0.03)	78.55%
transport	0.23	(0.04, 0.40)	99.38%	5.90e-03	(-0.03, 0.31)	62.07%
civic	0.02	(-0.15, 0.21)	59.08%	6.44e-03	(-0.07, 0.11)	61.73%
religious	0.05	(-0.10, 0.19)	75.42%	4.20e-03	(-0.06, 0.08)	63.12%
fraternal	0.01	(-0.12, 0.17)	56.80%	-0.01	(-0.09, 0.05)	75.15%
union	-0.13	(-0.27, 0.02)	94.70%	-0.01	(-0.12, 0.08)	71.18%
charitable	0.28	(0.07, 0.52)	99.80%	0.06	(-0.02, 0.27)	84.40%
phone	0.13	(-0.02, 0.30)	93.47%	-2.57e-03	(-0.05, 0.08)	61.97%
language	-0.04	(-0.29, 0.21)	62.95%	8.88e-04	(-0.07, 0.23)	52.72%
federal	-0.01	(-0.11, 0.07)	63.85%	3.12e-03	(-0.04, 0.05)	64.15%
state	6.48e-03	(-0.12, 0.10)	55.75%	-1.43e-03	(-0.07, 0.04)	58.82%
local	0.17	(0.00, 0.33)	97.55%	2.43e-04	(-0.04, 0.09)	51.72%
educ_eq	0.24	(0.06, 0.40)	99.48%	5.15e-03	(-0.03, 0.10)	69.63%
employ_eq	-0.09	(-0.26, 0.05)	91.40%	-9.90e-03	(-0.10, 0.05)	71.70%
ethnic_eq	-0.01	(-0.19, 0.11)	59.08%	-7.90e-04	(-0.07, 0.07)	55.20%
gender_inc	-0.06	(-0.21, 0.02)	92.05%	3.89e-04	(-0.07, 0.03)	54.43%
income_eq	0.02	(-0.08, 0.17)	60.50%	1.73e-03	(-0.03, 0.08)	61.03%
under65	-0.08	(-0.31, 0.03)	93.50%	4.96e-04	(-0.06, 0.05)	55.18%
civic_ops	0.04	(-0.06, 0.14)	83.97%	5.25e-03	(-0.03, 0.09)	72.25%
politic_act	-0.20	(-0.36, -0.05)	99.78%	-0.05	(-0.31, -0.00)	82.07%
over18	-0.27	(-0.51, -0.00)	98.17%	-3.78e-04	(-0.13, 0.06)	55.70%
**GROUP EFFECTS**						
Level.CNTY	-0.14	(-1.07, 1.02)	68.97%	-0.21	(-1.51, 1.09)	71.65%
Level.COMM	-0.13	(-1.08, 1.02)	68.97%	-0.23	(-1.50, 1.11)	72.67%
Level.FPRD	0.32	(-0.50, 1.62)	84.72%	0.46	(-0.67, 2.02)	86.40%
State.AZ				1.85	(0.66, 3.15)	99.85%
State.CA				1.19	(0.37, 2.01)	99.67%
State.CO				1.65	(0.79, 2.48)	99.98%
State.DE				-1.10	(-3.57, 0.89)	86.18%
State.FL				-1.78	(-3.42, -0.35)	99.28%
State.GA				-1.51	(-2.39, -0.65)	99.95%
State.IA				2.07	(-0.38, 5.60)	94.72%
State.ID				0.81	(-0.19, 1.80)	93.95%
State.IL				1.32	(-0.52, 3.59)	91.62%
State.IN				-2.42	(-5.64, -0.22)	98.42%
State.KY				-2.55	(-3.65, -1.51)	100%

	Model 1: No State			Model 2: State Fixed Effects		
**Parameter**	**Median**	**95% CI**	**pd**	**Median**	**95% CI**	**pd**
State.MA				1.07	(-0.83, 3.39)	86.30%
State.MD				-0.70	(-1.68, 0.26)	92.78%
State.ME				-2.52	(-5.84, -0.28)	98.82%
State.MI				0.47	(-1.30, 2.28)	69.78%
State.MN				2.57	(0.46, 5.67)	99.33%
State.MO				-1.74	(-5.54, 1.34)	86.57%
State.MS				-1.85	(-3.11, -0.75)	99.98%
State.MT				0.92	(-0.10, 1.91)	96.08%
State.NE				3.17	(1.24, 6.18)	100%
State.NH				-0.33	(-1.30, 0.59)	75.82%
State.NJ				-2.11	(-3.43, -0.96)	99.98%
State.NM				1.38	(0.40, 2.40)	99.57%
State.NV				3.00	(1.52, 5.02)	100%
State.NY				2.22	(-0.14, 5.70)	96.72%
State.OH				-2.28	(-4.42, -0.65)	99.75%
State.OK				-2.24	(-5.52, 0.08)	96.92%
State.OR				0.38	(-0.51, 1.25)	80.87%
State.PA				-0.29	(-2.27, 1.77)	61.98%
State.RI				-1.35	(-5.09, 1.72)	80.78%
State.SD				1.62	(-0.15, 3.86)	96.02%
State.TN				-1.64	(-5.22, 0.95)	88.68%
State.TX				1.44	(0.45, 2.46)	99.75%
State.UT				1.80	(0.91, 2.68)	100%
State.VA				1.14	(-1.98, 4.81)	76.47%
State.VT				0.35	(-1.90, 2.82)	62.03%
State.WA				0.65	(-0.28, 1.56)	91.78%
State.WI				-0.83	(-2.03, 0.32)	91.95%
State.WV				-3.55	(-6.43, -1.69)	100%
State.WY				0.03	(-0.96, 1.03)	52.47%
Year.2002	9.27e-03	(-0.37, 0.49)	54.97%	0.04	(-0.57, 0.74)	57.27%
Year.2003	-0.04	(-0.58, 0.30)	62.30%	-0.06	(-0.70, 0.45)	60.03%
Year.2004	-0.05	(-0.48, 0.20)	67.55%	-0.06	(-0.53, 0.38)	60.50%
Year.2005	0.09	(-0.13, 0.52)	77.33%	0.25	(-0.14, 0.76)	88.42%
Year.2006	-0.05	(-0.43, 0.21)	67.83%	-0.17	(-0.63, 0.22)	80.65%
Year.2007	0.07	(-0.17, 0.45)	71.55%	0.15	(-0.22, 0.61)	77.50%
Year.2008	0.03	(-0.21, 0.35)	63.55%	0.21	(-0.17, 0.68)	84.52%
Year.2009	-0.03	(-0.36, 0.23)	63.38%	-0.19	(-0.64, 0.18)	83.37%
Year.2010	-0.06	(-0.49, 0.18)	70.90%	-0.24	(-0.76, 0.17)	87.23%
Year.2011	0.08	(-0.16, 0.47)	73.65%	0.21	(-0.18, 0.71)	84.37%
Year.2012	-0.02	(-0.37, 0.26)	57.77%	0.03	(-0.40, 0.50)	56.98%
Year.2013	1.35e-03	(-0.33, 0.32)	50.78%	0.04	(-0.41, 0.55)	57.98%
Year.2014	-4.09e-03	(-0.33, 0.30)	52.58%	0.12	(-0.29, 0.59)	72.17%
Year.2015	0.08	(-0.17, 0.54)	74.05%	0.23	(-0.19, 0.81)	85.00%
Year.2016	-0.04	(-0.40, 0.22)	64.62%	-0.19	(-0.64, 0.17)	84.70%
Year.2017	3.54e-04	(-0.31, 0.32)	50.10%	0.05	(-0.34, 0.48)	62.03%
Year.2018	-0.03	(-0.36, 0.23)	63.00%	3.31e-03	(-0.40, 0.41)	50.90%
Year.2019	7.83e-03	(-0.27, 0.33)	53.70%	-9.31e-03	(-0.42, 0.39)	52.58%
Year.2020	0.18	(-0.05, 0.70)	87.65%	0.21	(-0.17, 0.72)	85.37%
Year.2021	-0.08	(-0.49, 0.16)	74.80%	-0.42	(-1.00, 0.03)	95.97%
Year.2022	-0.02	(-0.41, 0.27)	58.45%	-0.10	(-0.61, 0.34)	68.73%
Year.2023	-0.08	(-0.52, 0.15)	75.92%	-0.24	(-0.76, 0.17)	86.05%
Year.2024	0.03	(-0.25, 0.41)	62.48%	0.08	(-0.36, 0.59)	63.72%

Table S7. Zero-inflated beta regression predicting similarity between values stated in CWPPs and in federal policies

**Policy Values Only**	**Model 1: State FE**		**Model 2: State FE, interactions in σ**		**Model 3: State RE**		**Model 4: State RE, interactions in σ**	
Beta component (μ)	***β***	***sig***	***β***	***sig***	***β***	***sig***	***β***	***sig***
(Intercept)	-1.121	***	-1.125	***	-0.927	***	-0.930	***
Policy_CommunityForestRestorationAct	-0.321	***	-0.319	***	-0.320	***	-0.319	***
Policy_CohesiveStrategy	0.220	***	0.220	***	0.224	***	0.224	***
Policy_FLAMEAct	-0.003		0.000		-0.003		0.000	
Policy_FLRA	0.276	***	0.274	***	0.276	***	0.274	***
Policy_HFRA	-0.107		-0.144		-0.107		-0.144	
Policy_SharedStewardshipStrategy	0.041		0.043		0.042		0.044	
Policy_StewardshipContracting	0.205	***	0.208	***	0.205	***	0.208	***
Policy_WCF	-0.212	***	-0.213	***	-0.211	***	-0.213	***
Policy_WCIP	0.382	***	0.384	***	0.376	***	0.379	***
Policy_WildfireRiskReduction	-0.046		-0.042		-0.049	*	-0.045	
Policy_WydenAmendment	0.028		0.030		0.028		0.030	
Timing_Post	0.026		0.027		0.025		0.026	
Level_COMM	-0.068	***	-0.065	***	-0.071	***	-0.069	***
Level_FPRD	-0.010		-0.009		-0.012		-0.011	
Policy_CohesiveStrategyPhaseII:Timing_Post	0.181	***	0.186	***	0.178	***	0.183	***
Policy_FLAMEAct:Timing_Post	0.065		0.063		0.063		0.061	

Policy_FLRA:Timing_Post	-0.256	***	-0.251	***	-0.255	***	-0.250	***
Policy_HFRA:Timing_Post	-0.015		0.024		-0.015		0.024	
Policy_SharedStewardshipStrategy:Timing_Post	-0.067		-0.071		-0.068		-0.072	
Policy_StewardshipContracting:Timing_Post	-0.261	***	-0.268	***	-0.260	***	-0.267	***
Policy_WCF:Timing_Post	0.032		0.037		0.034		0.040	
Policy_WCIP:Timing_Post	0.246	***	0.299	***	0.237	***	0.289	***
Policy_WildfireRiskReduction:Timing_Post	0.005		-0.003		0.003		-0.005	
Timing_Post:Level_COMM	-0.005		-0.009		-0.002		-0.006	
Timing_Post:Level_FPRD	0.056		0.053		0.056	*	0.053	
Zero-inflation component (σ)	***γ***	***sig***	***γ***	***sig***	***γ***	***sig***	***γ***	***sig***
(Intercept)	3.042	***	3.035	***	3.046	***	3.040	***
Policy_CommunityForestRestorationAct	0.297	***	0.287	***	0.294	***	0.283	***
Policy_CohesiveStrategyPhaseII	-0.332	***	-0.320	***	-0.328	***	-0.316	***
Policy_FLAMEAct	-0.186	**	-0.218	*	-0.183	***	-0.216	*
Policy_FLRA	-0.421	***	-0.374	***	-0.424	***	-0.376	***
Policy_HFRA	0.045		0.830		0.042		0.832	
Policy_SharedStewardshipStrategy	-0.148	**	-0.172	*	-0.151	**	-0.174	*
Policy_StewardshipContracting	-0.416	***	-0.447	***	-0.418	***	-0.448	***
Policy_WCF	0.113		0.144		0.106		0.144	
Policy_WCIP	-0.328	***	-0.283	***	-0.315	***	-0.271	***
Policy_WildfireRiskReduction	-0.005		-0.020		0.006		-0.009	

Policy_WydenAmendment	-0.055	-0.066	-0.058	-0.070
Timing_Post	-0.068 *	-0.050	-0.068 *	-0.046
Level_COMM	-0.018	-0.012	-0.011	-0.003
Level_FPRD	-0.044	-0.075	-0.036	-0.065
Policy_CohesiveStrategyPhaseII:Timing_Post		-0.026		-0.024
Policy_FLAMEAct:Timing_Post		0.039		0.041
Policy_FLRA:Timing_Post		-0.076		-0.078
Policy_HFRA:Timing_Post		-0.799		-0.804
Policy_SharedStewardshipStrategy:Timing_Post		0.115		0.110
Policy_StewardshipContracting:Timing_Post		0.079		0.076
Policy_WCF:Timing_Post		-0.065		-0.080
Policy_WCIP:Timing_Post		-0.659 ***		-0.641 ***
Policy_WildfireRiskReduction:Timing_Post		0.208		0.210
Timing_Post:Level_COMM		-0.013		-0.018
Timing_Post:Level_FPRD		0.057		0.053
Global Deviance	***-1094.3***	***-1128.9***	***-1083.5***	***-1118.6***
AIC	***-846.3***	***-858.9***	***-853.3***	***-865.9***
SBC	***146.7***	***222.2***	***68.4***	***146.0***

** p < 0.05; ** p < 0.01; *** p < 0.001*

Table S8. Local context variables

Symbol	Variable	Definition
**Wildfire risk** (Source: Wildfire Risk to Communities Spatial Datasets (2))		
Exposure	Exposure type	For every pixel on the landscape, this layer delineates whether a structure at that location would be directly exposed to wildfire from adjacent wildland vegetation, indirectly exposed to wildfire from indirect sources such as embers and home-to-home ignition, or not exposed to wildfire due to distance from direct and indirect ignition sources
Fire_Intense	Fire intensity	An index comprised of Conditional Risk to Potential Structures (the potential consequences of fire to a home at a given location, if a fire occurs there and if a home were located there); Conditional Flame Length (Most likely flame length at a given location if a fire occurs, based on all simulated fires); Flame Exceedance Length 4' (Probability of having flame lengths greater than 4 feet if a fire occurs); and Flame Exceedance Length 8' (Probability of having flame lengths greater than 8 feet if a fire occurs)
Fire_Risk	Wildfire risk	An index comprised of Burn Probability (The annual probability of wildfire burning in a specific location) and Risk to Potential Structures (A measure that integrates wildfire likelihood and intensity with generalized consequences to a home on every pixel)
WHP	Wildfire hazard potential	An index that quantifies the relative potential for wildfire that may be difficult to control, used as a measure to help prioritize where fuel treatments may be needed
**Hazard Exposure** (Source: National Risk Index (3))		
ALR_VALA	Annual loss rate: agriculture	The proportion of the total value expected to be lost annually for a given community due to natural hazard impacts on agriculture
ALR_VALB	Annual loss rate: buildings	The proportion of the total value expected to be lost annually for a given community due to natural hazard impacts on buildings
ALR_VALP	Annual loss rate: population	The proportion of the total value expected to be lost annually for a given community due to natural hazard impacts on the population of an area
PM25	Particulate matter exposure	The amount of PM2.5 exposure (IDW-based)
**Social Vulnerability** (Source: Climate & Environmental Justice Screening Tool (4))		
diabetes	Percent with diabetes	Share of people ages 18 years and older who have been told by a health professional that they have diabetes other than diabetes during pregnancy
asthma	Percent with asthma	Share of people who answer "yes" to both of these questions: "Have you ever been told by a health professional that you have asthma?" and "Do you still have asthma?"

Symbol	Variable	Definition
expect	Life expectancy	Average number of years people have left in their lives
houseburden	Housing burden	Share of households that are both earning less than 80% of Housing and Urban Development's Area Median Family Income and are spending more than 30% of their income on housing costs
plumbing	Plumbing access	Share of households without indoor kitchen facilities or complete plumbing facilities
transport	Transportation access	Average relative cost and time spent on transportation relative to all other tracts
income	Household median income	Household median income at the tract-level circa 2019
unemployed	Proportion unemployed	Number of unemployed people as a share of the labor force at the block-group circa 2019
poverty	Proportion in poverty	Share of people living at or below 100% of the Federal poverty level at the tract-level circa 2019
language	Language competency	% of total population proficient English speakers
phone	Communication capacity	% of households with a telephone
**Social Capital** (Source: Kyne & Aldrich Social Capital Index (5))		
religious	Religious organizations	Number of religious organizations per 10,000 people
civic	Civic organizations	Number of civic organizations per 10,000 people
charitable	Charitable connections	Percent of total population that are members of charitable organizations
fraternal	Fraternal organization connections	Percent of total population that are members of fraternal organizations
union	Union connections	Percent of total population that are members of unions
over18	Political linkage	Percent of total population that are eligible for voting
local	Local government linkage	Percent of total population working for local governments
state	State government linkage	Percent of total population working for state governments
federal	Federal government	Percent of total population working for federal governments

Symbol	Variable	Definition
	linkage	
politic_act	Political acts	Percent of total population that participated in a political rally, speech, or organized protest
ethnic_eq	Race similarity	Race fractionation (the fuzzy sum of proportion of the population comprised by each Census-defined race)
educ_eq	Educational equality	Negative absolute difference between % of total population with college education and % of total population with less than high school education
income_eq	Income equality	Gini coefficient (0=perfect equality to 1=perfect inequality)
employ_eq	Employment equality	Absolute difference between % of total employed and % of total unemployed labor force
gender_inc	Gender-income Similarity	Gender income fractionalization (0=complete homogeneity to 1=complete heterogeneity)
under65	Non-elder population	Percent of total population under 65
civic_ops	Civic opportunities	Civic opportunities index (6)

Table S9. List of policies

Name	Shorthand Name	Year	Reference/Author
A National Cohesive Wildland Fire Management Strategy Phase II Report	Cohesive Strategy	2012	Wildland Fire Leadership Council (USDA and DOI) (7)
Community Forest Restoration Act	CFRA	2000	S. 1288
Federal Land Assistance, Management and Enhancement Act	FLAME Act	2009	HR 110-704
Forest Landscape Restoration Act	FLRA	2008	SR 110-370
Healthy Forests Restoration Act	HFRA	2003	PL 108-148, as amended by PL 117-328
National Forest System Land Management Planning	2012 Planning Rule	2012	36 CFR Part 219
Stewardship end result contracting projects	Stewardship Contracting	2014	16 USC § 6591c
Toward Shared Stewardship Across Landscapes: An Outcome-Based Investment Strategy	Shared Stewardship Strategy	2018	USDA FS-1118
Watershed Condition Framework	WCF	2011	USDA FS-977
Wildfire Crisis Implementation Plan	WCIP	2022	USDA FS-1187b
Wildfire Risk Reduction		2021	16 USC § 6592
Wyden Amendment		1998	PL 105-277 § 323, as amended by PL 109-54 § 434

Note: USDA = United States Department of Agriculture. DOI = Department of the Interior.

SI References

1. P. J. Jakes, *et al.*, Community wildfire protection planning: is the Healthy Forests Restoration Act's vagueness genius? *Int J Wildland Fire* **20**, 350–363 (2011).
2. J. H. Scott, *et al.*, Wildfire Risk to Communities: Spatial datasets of landscape-wide wildfire risk components for the United States (2nd Edition). Forest Service Research Data Archive. https://doi.org/10.2737/RDS-2020-0016-2. Deposited 2024.
3. National Risk Index. Available at: https://hazards.fema.gov/nri/ [Accessed 8 July 2025].
4. Climate + Economic Justice Screening Tool. *Data + Screening Tools* (2025). Available at: https://screening-tools.com/climate-economic-justice-screening-tool [Accessed 5 August 2025].
5. D. Kyne, D. P. Aldrich, Capturing Bonding, Bridging, and Linking Social Capital through Publicly Available Data. *Risk, Hazards & Crisis in Public Policy* **11**, 61–86 (2020).
6. M. de Vries, J. Y. Kim, H. Han, The unequal landscape of civic opportunity in America. *Nature Human Behaviour* **8**, 256–263 (2023).
7. Wildland Fire Leadership Council, "A National Cohesive Wildland Fire Management Strategy: Phase II National Report" (2012).
